# SA-β-Gal stratification reveals senescence-associated heterogeneity and differential cell death susceptibility in human CD8 effector T cells

**DOI:** 10.3389/fimmu.2026.1985303

**Published:** 2026-10-26

**Authors:** Luis Garza-Martínez, Mark Apadeer, Nica Mariz Aquino, Paolo S. Turano, Elizabeth Akbulut, Dillon Oswald, Sukhwinder Singh, Patricia Fitzgerald-Bocarsly

**Affiliations:** 1 Rutgers School of Graduate Studies, Rutgers Biomedical and Health Sciences, Rutgers University, Newark, NJ, United States; 2 Department of Pathology, Immunology, and Laboratory Medicine, Rutgers-New Jersey Medical School, Newark, NJ, United States; 3 Department of Microbiology, Biochemistry and Molecular Genetics, Center for Cell Signaling, Rutgers-New Jersey Medical School, Newark, NJ, United States

**Keywords:** CD8 T cell, cell death, effector memory T cell, EMRA T cells, granzymes, nutrient limitation, senescence-associated β-galactosidase (SA-β-Gal), T-cell senescence

## Abstract

**Introduction:**

Cellular senescence in human T cells remains incompletely defined, particularly with respect to how senescence-associated states influence susceptibility to cell death.

**Methods:**

We used flow-cytometric SPiDER-based detection of senescence-associated β-galactosidase (SA-β-Gal) activity to profile human CD8+ T-cell subsets from young and old donors. We stratified effector memory (CD8_EM_) and effector memory re-expressing CD45RA (CD8_EMRA_) subsets by the lowest (bottom 25%) and highest (top 25%) quartiles of SA-β-Gal signal.

**Results:**

CD8_EM_ and CD8_EMRA_ cells displayed the greatest within-subset heterogeneity in SA-β-Gal activity. The SA-β-Gal low and high populations occupied distinct regions in UMAP projections and were reproducibly enriched in different FlowSOM metaclusters, indicating that SA-β-Gal activity captures structured phenotypic states within differentiated CD8+ T-cell compartments. These states differed in senescence-associated markers, including CD27, CD28, CD57, and CX3CR1, as well as granzyme-associated programs involving Gzm A, B and K, whereas chronological age had only a limited effect once subsets were analyzed separately. Functionally, nutrient-limited conditions unmasked marked differences in cell death susceptibility between these populations. Following PMA/ionomycin stimulation, SA-β-Gal high CD8_EM_ and CD8_EMRA_ cells underwent accelerated stimulation-induced cell death, showed increased caspase-3/7 activation, and exhibited lower post-treatment γH2AX signal relative to SA-β-Gal low counterparts. In contrast, in the absence of stimulation-induced energetic demand, SA-β-Gal high cells displayed greater resistance to cell death.

**Discussion:**

Together, SA-β-Gal stratification resolves structured heterogeneity within human CD8 effector T cells and supports a context-dependent model in which senescence-associated effector states become selectively vulnerable to stimulation-induced death under metabolic stress.

## 1 Introduction

Following activation, circulating naïve CD8+ T cells differentiate into memory subsets that differ in longevity, proliferative capacity, and functional potential (1–3). These CD8+ T cell subsets are organized into discrete differentiation states, including central memory (CD8_CM_), effector memory (CD8_EM_), and effector memory re-expressing CD45RA (CD8_EMRA_). The proliferative potential and longevity of human CD8+ T cell subsets vary significantly depending on the experimental model and the disease context, with naïve CD8 T cells being exceptionally long-lived. Isotope-labeling studies have estimated the half-life of naïve CD8 T cells at approximately 6.5 years, with estimates ranging from 6 to 10 years (4, 5). In contrast, this same isotope-labeling study estimated memory CD8 T cell half-life of approximately 244 days (4). Another study showed that antigen-specific memory CD8 populations can persist for at least a decade and divide less than once per year after yellow fever vaccination (6). Within T cell subsets, the proliferative capacity mirrors their persistence, as naïve cells retain the greatest proliferative reserve, whereas T_EMRA_ show the highest ex vivo BrdU turnover among CD45RA/CCR7-defined CD8 subsets (7, 8). In terms of functional potential of these subsets, naïve cells lack immediate effector function but retain developmental potential. CD8_CM_ show limited immediate cytotoxicity but preserve recall capacity and can differentiate into effector-like cells. CD8_EM_ and CD8_EMRA_ are enriched for immediate effector cytokine and cytotoxic programs characterized by granzyme B and perforin expression (7, 9, 10). The influence of the immunological memory process is also reflected in the acquisition of senescence-associated features. As human CD8 T cells progress from naïve/CM toward EM and late EMRA-like states, they downregulate CD28/CD27 and upregulate CD57 expression, and increasingly acquire senescence-associated features, especially, telomere erosion, impaired proliferation, reduced telomerase inducibility, and a pro-inflammatory phenotype (11–14). However, cellular senescence in human T cells remains incompletely defined and it is still unclear whether the acquisition of these senescence-associated features within differentiated CD8+ T cell subsets represent a uniform state or instead resolve into functionally distinct subpopulations.

Previous work from the Herbig and Fitzgerald-Bocarsly laboratories established that human CD8+ T cells with high SA-β-Gal activity display senescence-associated molecular and functional features and showed that these transcriptional programs are largely preserved across chronological age (15, 16) in that CD8+ T cells with high levels of SA-β-Gal activity could be found both in younger and older donors (albeit at lower frequency in the younger donors), but that these high SA-β-Gal cells were transcriptionally very similar across ages. Although CD8+ T cell subsets are often treated as homogeneous, transcriptional and phenotypic analyses have revealed substantial diversity within these subsets, particularly in the EMRA population (11, 17–19). CD28, CD57 and CD27 have been identified as senescence-associated markers that can resolve the population heterogeneity that exists within human CD8_EMRA_ in the context of senescence. Additionally, p38 MAPK has been identified as the regulatory pathway of the senescence-associated secretory phenotype (SASP) that the CD27− EMRA population exhibits. Together, these observations support the idea that T-cell senescence may exist, at least in part, along a continuum that emerges from the memory differentiation program rather than a wholly distinct fate (20). Consistent with this view, using a mammary epithelial cell model, Ashraf et al. showed that canonical senescence biomarkers, including SA-β-Gal activity, can behave as graded rather than binary readouts (21). We selected flow-cytometric SPiDER-βGal detection because it enables the use of flow cytometry for quantitative single-cell measurement of SA-β-Gal activity alongside surface-marker phenotyping and allows isolation of viable populations by cell sorting for subsequent functional assays; we have previously published using this methodology and showed it marked the same cells as conventional histochemical detection methodology (15). These capabilities motivated its use over conventional histochemical SA-β-Gal staining for investigating heterogeneity within defined CD8+ T-cell subsets. Following this rationale, rather than using SA-β-Gal activity as a standalone definition of senescence, we used it to resolve heterogeneity within differentiated CD8+ T-cell subsets, building on its association with senescence-related molecular and functional features in our previous studies (15, 16). To do this we used a quartile based approach gating within CD8+ T cell subsets where we defined the cells with the lowest (bottom 25%) and highest (top 25%) SA-β-Gal activity as “SA-β-Gal low” and “SA-β-Gal high”, respectively. These designations describe relative SA-β-Gal activity within each donor and subset and do not constitute validated thresholds separating senescent from nonsenescent cells.

An important unresolved question in the T cell senescence field is how T cell senescence influences susceptibility to apoptosis. In fibroblasts, senescent cells were shown to fail to suppress pro-survival programs involving genes such as Bcl2 and to develop resistance to apoptosis (22, 23). Later studies found that this phenomenon could be also observed in CD8+ T cells that have reached replicative senescence (24). However, it remains uncertain whether this framework applies to human T cells under conditions that resemble those of activation or the metabolic stress induced by limited nutrient availability, such as that observed in the tumor microenvironment. This question is especially relevant in effector T cells, in which activation-induced cell death helps constrain the magnitude, the duration and by extension, the efficacy of the immune response. In this context, a key unresolved issue is not only whether senescence-associated states can be identified within effector subsets, but whether these cells differ in their ability to survive activation under metabolic constraint. In this study, we observed that CD8_EM_ and CD8_EMRA_ cells displayed the greatest heterogeneity in SA-β-Gal activity at the subset level and we reasoned that stratifying effector subsets according to SA-β-Gal activity could resolve different functional states within differentiated human CD8+ T cells. We therefore tested whether populations with **SA-β-Gal high and SA-β-Gal activity** within CD8_EM_ and CD8_EMRA_ cells differ in their susceptibility to stimulation-induced cell death and the DNA damage-response (DDR) signaling under nutrient-limited conditions by measuring caspase-3/7 activation, phosphatidylserine externalization, loss of plasma membrane integrity, and γH2AX induction.

## 2 Methods

### 2.1 Peripheral blood mononuclear cell isolation

The Institutional Review Board of New Jersey Medical School approved this study (ID Pro0119980237). Peripheral blood was obtained from healthy young donors (20–34 years old) and old donors (>60 years old) after informed consent and collected in heparinized tubes. Older donors were recruited through the Aging & Brain Health Alliance (ABHA), a university–community partnership established in 2006 to support community engagement and recruitment into biomedical research at Rutgers University–Newark. Eligible participants were aged 60 years or older, self-identified as African American or Black, could speak, read, and understand English, and were willing to provide a blood sample for immunological analyses. Participants were excluded from the current analyses if they had a Montreal Cognitive Assessment (MoCA) score ≤18; a diagnosed neurodegenerative disorder or learning disability; a history of major stroke or serious brain injury; current use of medications typically prescribed for dementia (e.g., Aricept, Exelon, or Namzaric); self-reported excessive alcohol or recreational drug use; a medical procedure requiring general anesthesia within the preceding 3 months; or color blindness. CMV serostatus was not confirmed for the young or older donors, except for the commercially obtained samples used in **Supplementary Figures 1B–D**, for which CMV serostatus was provided by the supplier. Peripheral blood mononuclear cells (PBMCs) were isolated by density-gradient centrifugation using Lymphocyte Separation Medium (Corning, Manassas, VA) according to the manufacturer’s instructions. PBMCs were cultured in RPMI 1640 (VWR Life Science, Radnor, PA) supplemented with L-glutamine (Corning), 10% heat-inactivated fetal bovine serum (Gibco, Gaithersburg, MD), 100 U/ml penicillin, 100 ug/ml streptomycin, 100 ug/ml gentamicin, and 5 mM HEPES (Sigma Aldrich, St. Louis, MO). Following PBMC isolation, cells were counted and assessed for viability using a Cellometer Auto 2000 (Nexcelom, Lawrence, MA) with ViaStain AOPI Staining Solution (Revvity Health Sciences, Hopkinton, MA). Cell viability was ≥97% at the start of the experiments, and cells were cultured at 2 × 10^6^ cells/mL.

### 2.2 Surface antibodies and SPiDER staining reagents

For CD8+ T-cell subset identification, the following antibodies were used: Pacific Blue anti-CD3 (UCHT1), PerCP-Cy5.5 anti-CD3 (HIT3a), PE-Cy7 anti-CD4 (SK3/OKT), APC anti-CD8 (HIT8a), PE anti-CD8 (RPA-T8), APC-Cy7 anti-CCR7 (G043H7), BV785 anti-CCR7 (G043H7), and BV711 anti-CD45RA (HI100) (all from BioLegend, San Diego, CA). For senescence-associated phenotyping, the following antibodies were used: BV510 anti-CD57 (QA17A04), BV421 anti-CD27 (0323), APC-Fire anti-CD28 (CD28.2), and APC anti-CX3CR1 (2A9-1) (BioLegend). Senescence-associated β-galactosidase activity was detected using the Cellular Senescence Detection Kit SPiDER-βGal (Dojindo Molecular Technologies, Rockville, MD).

### 2.3 Surface staining-SPiDER protocol

For SA-β-Gal detection, bafilomycin A1 and SPiDER-βGal (Dojindo, catalogue no. SG04-10) stock solutions were prepared by reconstituting the product in 30 μl and 20 μl DMSO, respectively. PBMCs were incubated with the stock solution of bafilomycin A1 at a 1:500 dilution for 1 h, followed by the stock solution of SPiDER-βGal at a 1:500 dilution for 30 min. Cells were then washed with PBS and stained with surface antibodies for 20 min at 4 °C. After washing, cells were resuspended in DAPI (25 μl/ml; BioLegend) immediately before acquisition or sorting to exclude dead cells. Samples were acquired on a FACSymphony A5 SE flow cytometer (BD Biosciences, Franklin Lakes, NJ), collecting 3 × 10^5^ to 5 × 10^5^ events per sample, and were analyzed using FlowJo software.

### 2.4 Fixation/permeabilization

Cells were fixed with 4% paraformaldehyde for 5 min at 4 °C, washed with PBS, and permeabilized with 0.2% Triton X-100 in PBS for 10 min at room temperature. After permeabilization, cells were washed with PBS and processed for intracellular staining.

### 2.5 Granzyme staining

PBMCs (2 × 10^6^) were stained with bafilomycin A1, SPiDER-βGal, and surface antibodies as described above. Cells were then fixed, permeabilized, and stained intracellularly for 30 min with Granzyme A-AF594 (BioLegend, San Diego, CA), Granzyme B-Pacific Blue (BioLegend), and Granzyme K-PerCP-eFluor 710 (Invitrogen, Carlsbad, CA). After washing, cells were resuspended in PBS and acquired on a FACSymphony A5 SE flow cytometer.

### 2.6 Flow cytometry gating strategy

A lymphocyte gate was drawn from the PBMC population and CD8 T-cell subsets were identified according to CD8, CD3, CD45RA and CCR7 expression. To minimize cross-contamination between phenotypically adjacent subsets, CD45RA and CCR7 gates were drawn conservatively using histogram-defined peaks, and events falling between clearly resolved positive and negative populations were excluded from subset assignment. This was particularly important for separation of CD8 naïve and CD8_EMRA_ cells, whose close proximity in conventional quadrant gating can introduce naïve-cell contamination into EMRA gates and artificially broaden or distort SPiDER distributions.

### 2.7 Processing and analysis of public scRNA-seq datasets

Publicly available single-cell RNA-seq data from Favaloro et al. (25) were used for comparative visualization of gene-expression patterns relevant to the present study. Published differential-expression results for CD8+ T-cell populations from healthy controls and patients with multiple myeloma were extracted from the dataset and visualized as volcano plots in GraphPad Prism.

### 2.8 CD8+ T cell enrichment and cell sorting

CD8+ T cells were enriched from PBMCs by negative selection using the EasySep Human CD8+ T Cell Isolation Kit (STEMCELL Technologies, Cambridge, MA) according to the manufacturer’s instructions. Purity was assessed by flow cytometry as the percentage of CD3+CD8+ cells and ranged from 95% to 98%. Enriched cells were counted using a Cellometer Auto 2000 and sorted on a BD FACSAria Fusion (BD Biosciences, Franklin Lakes, NJ). Purified CD8+ T cells were pretreated with bafilomycin A1, which remained present during the subsequent SPiDER-βGal incubation. Following SPiDER staining, cells were washed once with PBS and stained with surface antibodies as described above. For subset-specific experiments, CD8_EM_ and CD8_EMRA_ cells were first gated and then sorted into SA-β-Gal low and SA-β-Gal high fractions corresponding to the bottom and top quartiles of SPiDER fluorescence within each subset.

### 2.9 H2AX DNA-damage staining

PBMCs (2 × 10^6^) were stained with bafilomycin A1, SPiDER-βGal, and surface antibodies as described above. Cells were then stimulated with PMA (500 ng/ml) plus ionomycin (500 ng/ml) or treated with H_2_O_2_ (50 μM) for 30 min at 37 °C. After treatment, cells were fixed in 4% paraformaldehyde for 5 min at 4 °C, permeabilized with 0.2% Triton X-100 for 10 min at room temperature, and stained intracellularly for 30 min at 4 °C with PE-conjugated phospho-Histone H2A.X (Ser139) antibody (clone N1-431; BD Biosciences). Cells were washed, resuspended in PBS, and acquired on a FACSymphony A5 SE flow cytometer.

### 2.10 CMV tetramer assay

PBMCs (3 × 10^6^) from HLA-A02:01-positive CMV-seropositive or CMV-seronegative donors (Cytologics Inc., Brighton, NY) were stained with bafilomycin A1 and SPiDER-βGal as described above. Cells were then incubated for 30 min at 4 °C with pp65 (NLVPMVATV)-HLA-A02:01 PE tetramer (0.1 μl at 1.34 mg/ml) together with antibodies used to define CD8+ T-cell subsets. Cells were fixed in 4% paraformaldehyde for 5 min at 4 °C, washed, resuspended in PBS, and acquired on a FACSymphony A5 SE flow cytometer.

### 2.11 *In vitro* cell death assay-flow cytometry

PBMCs (2 × 10^6^) were pretreated with bafilomycin A1 and stained with antibodies to identify CD8+ T-cell subsets as described above. Cells were then stimulated with PMA (500 ng/ml) plus ionomycin (500 ng/ml) or treated with H_2_O_2_ (50 μM) for 5 and 30 min at 37 °C. Early apoptosis was assessed by phosphatidylserine exposure using ApoTracker (BioLegend, San Diego, CA), and loss of plasma membrane integrity was assessed using Zombie-PE (BioLegend). Cells were incubated with these reagents for 25 min at room temperature, then fixed in 4% paraformaldehyde for 30 min at 4 °C, washed, stained with SPiDER-βGal for 30 min, washed again, resuspended in PBS, and acquired on a FACSymphony A5 SE flow cytometer.

### 2.12 *In vitro* cell death assay-time-lapse live-cell imaging assay

#### 2.12.1 Bulk CD8+ T cells

For bulk CD8+ T-cell assays, negatively selected CD8+ T cells were resuspended at 5 × 10^5^ cells/ml in HBSS. Then, 5 × 10^4^ cells were plated in technical duplicates in Falcon 48-well plates (Corning Life Sciences, Corning, NY) in the presence of 5 μM Caspase-3/7 Green Dye (Sartorius, item 4440), 1 μM Cytotox Red (Sartorius, item 4632), and PMA plus ionomycin (500 ng/ml each), where indicated. Images were acquired every 10–20 min for up to 24 h using the IncuCyte live-cell imaging platform, collecting four images per well at each time point. Exposure times were 200 ms for the green channel and 400 ms for the red channel. The percentage of dead cells for every timepoint was calculated using the following formula: $\%=\frac{Positive\;Events}{Total\;Events}\times 100$. Error bars represent the standard deviation (individual donors) or standard error of the mean (multiple averaged donors) of technical replicates used for each assay.

#### 2.12.2 CD8+ T cell low and high SA-β-Gal effector subsets

PBMCs were isolated as described above. CD8+ T cells were enriched, stained with antibodies against CD4, CD8, CD45RA, and CCR7, and treated with bafilomycin A1 and SPiDER-βGal as described above. CD8_EM_ and CD8_EMRA_ cells were then sorted into SA-β-Gal low and SA-β-Gal high fractions corresponding to the bottom and top quartiles of SPiDER fluorescence within each subset. Sorted cells (5 × 10^4^ per condition) were plated in technical duplicate in Falcon 48-well plates in the presence of 5 μM Caspase-3/7 Green Dye plus 1 μM Cytotox Red, or 1 μg/ml propidium iodide alone, with or without PMA plus ionomycin (500 ng/ml each). Images were acquired every 15 min for up to 24 h using the IncuCyte platform, collecting four images per well at each time point. Exposure times were 200 ms for the green channel and 400 ms for the red channel. The percentage of dead cells or caspase 3/7 positive cells for every timepoint was calculated using the following formula: $\%=\frac{Positive\;Events}{Total\;Events}\times 100$. Error bars represent the standard deviation of technical replicates used for each assay.

### 2.13 Estimated absolute counts

Estimated absolute numbers of CD4+ and CD8+ T-cell subsets were calculated by combining the absolute lymphocyte count from the CBC with subset frequencies measured by flow cytometry. For each donor, estimated absolute subset counts were calculated as: absolute subset count = absolute lymphocyte count × frequency of CD4+ or CD8+ T cells among lymphocytes × frequency of the indicated subset within CD4+ or CD8+ T cells. Because counting beads were not used, these values are reported as CBC-derived estimated absolute subset counts.

### 2.14 UMAP dimensionality reduction and visualization

UMAP embeddings were generated using the FlowJo UMAP plugin (v4.0.3) on compensated fluorescence channels corresponding either to the senescence-associated phenotyping panel or to the granzyme panel. The senescence-associated panel included CD27, CD28, CD57, and CX3CR1, whereas the granzyme panel included Gzm A, Gzm B, and Gzm K. UMAP was performed within pregated CD8+ T-cell subsets (naïve, EM, and EMRA) using concatenated events stratified by age group. SA-β-Gal was overlaid either as a continuous heat map or as categorical SA-β-Gal low and SA-β-Gal high fractions to visualize how SPiDER extremes mapped onto the phenotypic landscape.

### 2.15 Pearson correlation analysis

To assess co-expression relationships among markers, FCS files from bulk CD8+ T cells were concatenated across donors within each age group in FlowJo. Fluorescence intensity values for each marker were exported from the concatenated datasets, and pairwise Pearson correlation coefficients were calculated across single-cell events. Correlation matrices were visualized as heat maps. Pooled coefficients were used as descriptive summaries of the concatenated cell populations, without event-level significance testing. They were not used to establish the consistency of these associations across individual donors.

### 2.16 FlowSOM clustering and metacluster definition

Unsupervised clustering was performed using the FlowJo FlowSOM plugin (v4.1.0). To avoid circularity, SA-β-Gal was not included as an input channel for FlowSOM clustering. FlowSOM was trained on concatenated, per-donor downsampled events within each subset to ensure comparable donor contribution. Specifically, EM was downsampled to 2000 events per donor, EMRA to 1000 events per donor, and naive to 950 events per donor (one old donor displaying a CD8+ T cell count below this threshold was excluded). FlowSOM input channels were CD27, CD28, CD57, and CX3CR1 (compensated parameters). Self-organizing maps were trained using a 10 × 10 grid followed by metaclustering into 15 metaclusters. Trained maps were then applied to individual donor files and to SA-β-Gal High and SA-β-Gal Low quartile gates within each subset. To measure whether SA-β-Gal extremes occupy distinct phenotypic states, per-donor enrichment was computed for each metacluster as: ${\text{Δ}}_{k}=f_{k}\left(\text{High}\right)-f_{k}\left(\text{Low}\right),$ where *f_k_* is the frequency of metacluster *k* within SA-β-Gal High or SA-β-Gal Low, respectively. Positive Δ*_k_* values indicate enrichment in SA-β-Gal High, and negative values indicate enrichment in SA-β-Gal Low. Metacluster frequencies were computed as “frequency of parent” within each analyzed population.

To quantify donor-level phenotypic heterogeneity independent of SA-β-Gal stratification, Shannon entropy was computed from metacluster frequency vectors for each donor and subset. For a donor with metacluster proportions *p_k_* across *K* = 15 metaclusters, entropy was computed as:


$$H=-\sum_{k=1}^{K}p_{k}\log\left(p_{k}\right),\;$$

where *p_k_* values of 0 were excluded from the sum. Entropy was normalized to the range 0 to 1 as $H_{norm}=\frac{H}{\log\left(K\right)}$. Lower values indicate that a donor’s cells are concentrated into fewer metaclusters (lower phenotypic diversity), while higher values indicate broader distribution across metaclusters (higher phenotypic diversity).

### 2.17 mTOR assays

#### 2.17.1 Mitochondrial ROS production

1x10^6^ PBMCs were surface stained with CD8+ ab and stained with bafilomycin A1/Spider as described. Then PBMCs were pre-treated for 1h with 100nM/10 µM Rapamycin (MCE, Monmouth, NJ). After that, cells were simultaneously activated with 500 ng/ml PMA/Ionomycin (STEMCELL Technologies, Vancouver, Canada) and stained with mitochondrial ROS dye MitoBright ROS Deep Red (Dojindo Molecular Technologies, Inc, Rockville, MD) at 6000x final dilution for 30’ at 37 °C. Lastly, PBMCs were washed with PBS, then fixed as described and events recorded using a BD FACSymphony™ A5 flow cytometer (BD Biosciences, San Jose, CA).

#### 2.17.2 IncuCyte time-lapse live-cell imaging assay

Negatively enriched CD8+ T cells were resuspended at 5×10^5^ cells/ml in HBSS. Then, 5×10^4^ cells were plated in technical duplicate in Falcon 48-well plates (Corning Life Sciences, Corning, NY) and pretreated with 100nM/10 µM rapamycin for 1hr at 37 °C. Then 1 μg/ml propidium iodide with or without PMA plus ionomycin (500 ng/ml each) was added simultaneously and cells were placed in the IncuCyte chamber. Images were acquired every 10–20 min for up to 24h using the IncuCyte live-cell imaging platform, collecting four images per well at each time point. Exposure times were 200 ms for the green channel and 400 ms for the red channel. The percentage of dead cells for every timepoint was calculated using the following formula: $\%=\frac{Positive\;Events}{Total\;Events}\times 100$. Technical replicates were averaged within each donor. Error bars of individual donors represent the standard deviation of technical replicates whereas grouped donor summaries show the arithmetic mean ± SEM across independent donors.

#### 2.17.3 mTOR activation assay

2x10^6^ PBMCs were surface stained with CD8+ subset ab and stained with bafilomycin A1/Spider as described. Then, cells were resuspended in HBSS and pretreated for 1h at 37 °C with 100nM/10 µM Rapamycin. Then, cells were activated for 15’ at 37 °C with 500ng/ml PMA + 500ng/ml Ionomycin. Following activation, cells were washed with PBS, fixed and permed and stained with PE-Cy7-conjugated anti-phospho-S6 antibody targeting Ser235, Ser236 (cupk43k; Thermo Fisher Scientific, Waltham, MA) ab for 30’ at 4 °C.

## 3 Results

### 3.1 SA-β-Gal activity increases with differentiation and identifies maximal heterogeneity in CD8 effector subsets

Aging is associated with naïve T-cell attrition and relative enrichment of memory populations, especially in the CD8 compartment (26–28).Using our experimental design (**Figures 1A, B**), we first asked whether the apparent enrichment of effector populations reflected true expansion of those compartments or instead contraction of the naïve pool. To do this, we used flow cytometry to profile CD45RA- and CCR7-defined T cell subsets in PBMCs from young and old donors, and derived estimated absolute subset counts from CBC-based lymphocyte counts. Consistent with prior studies showing that naïve CD8+ T cells undergo the most pronounced age-associated decline among CD8+ T cell subsets (26, 29, 30), we observed that chronological aging was associated with reduced absolute CD8+, but not CD4+, T-cell counts, driven primarily by the contraction of the CD8+ naïve compartment (**Figures 1C–E**). Although the relative frequency of memory populations increased with age in both CD4+ and CD8+ lineages, this effect was most pronounced in the CD8+ compartment and largely reflected depletion of naïve cells rather than absolute accumulation or expansion of effector cells (**Figure 1E**). Thus, changes in subset composition provide an important framework for interpreting senescence-associated readouts in bulk T-cell populations. Although our data support the age-associated increase in the relative frequencies of CD8+ T cell memory subsets described by others (26, 29, 30), it is important to interpret them in the context of changes in absolute numbers. This distinction is important because age-associated increases in relative effector frequency can be misread as expansion of effector subsets, when they primarily reflect attrition of the naïve CD8+ pool.

**Figure 1:**
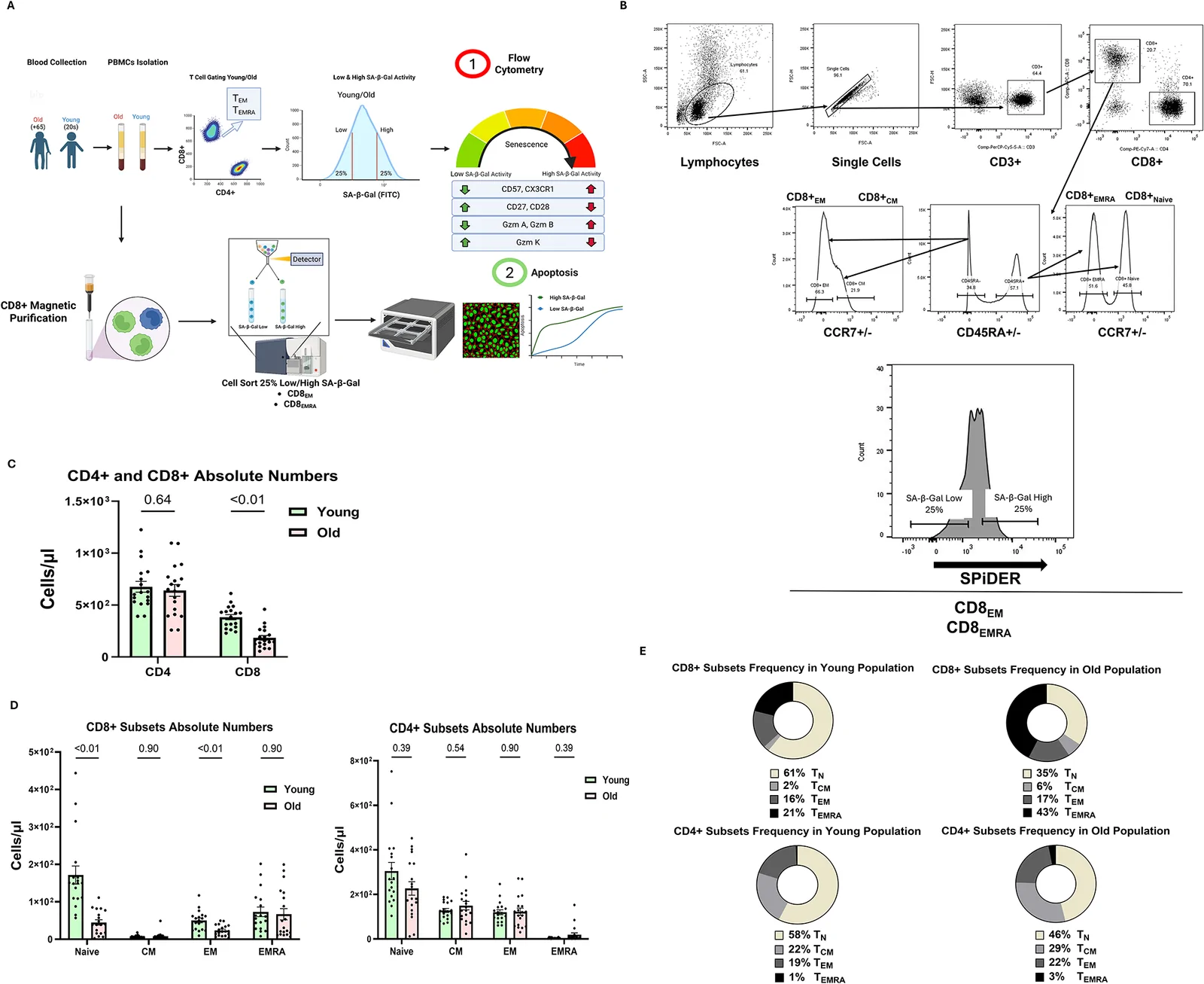
Chronological aging is associated with a significant reduction in the absolute number of CD8^+^ naïve T cells, resulting in a relative increase in the frequency of CD8^+^ memory subsets. **(A)** Experimental design for the characterization of CD8+ effector subsets. Figure created with BioRender.com. **(B)** Gating strategy used to identify the different CD8+ T cell subsets and to gate the SA-β-Gal high (top 25%) and SA-β-Gal low (bottom 25%) CD8_EM_ and CD8_EMRA_. **(C)** Whole blood counts (cells/ul) of CD4+ and CD8+ T cells in young (n=18) and old (n=19) donors **(D)** Whole blood counts (cells/ul) of CD4+ and CD8+ T cell subsets in young (n=18) and old (n=19) donors. **(E)** Donut charts from absolute numbers **(D)** show the relative frequency (percentage) of CD8+ and CD4+ subsets of young (n=18) and old donors (n=19). Statistical significance was determined by multiple unpaired t-tests with Holm-Šidák’s correction. Bars indicate mean ± SEM.

We next profiled SA-β-Gal activity across CD4+ and CD8+ T-cell subsets using SPiDER fluorescence as a flow-cytometric readout of SA-β-Gal activity. In agreement with our previous work, SA-β-Gal activity increased in bulk CD8+ and CD4+ T cells from older relative to younger donors (15) (**Figure 2A**). Because CD8+ T-cell subsets displayed distinct SPiDER fluorescence distributions, gates in the CD45RA/CCR7 space were drawn conservatively to minimize carryover between adjacent populations (**Figure 1B**). However, subset-resolved analysis showed that SA-β-Gal activity increased progressively with differentiation status, with naïve cells displaying the lowest signal and EMRA cells the highest in both CD8 and CD4 cells (**Figure 2B**). This finding complements our published results showing that the SA-β-Gal high fraction of bulk CD8+ T cells is enriched for differentiated subsets, whereas the SA-β-Gal low population was predominantly comprised of naïve cells (15). In contrast, chronological age had little effect within individual subsets, indicating that differentiation state, rather than age itself, is the dominant determinant of SA-β-Gal activity at the subset level. This applied across all subsets, but was particularly important for the naïve and EMRA compartments, which both contain CD45RA+ cells yet differ markedly in SPiDER fluorescence. In this setting, imprecise separation of these populations can artifactually broaden the EMRA SPiDER profile and create the appearance of bimodality through naïve-cell contamination. In this framework, the higher SA-β-Gal signal observed in bulk CD8+ T cells from older donors might best be interpreted as a consequence of age-associated remodeling toward a relative increase in the differentiated populations, rather than a uniform age-driven shift in SA-β-Gal activity within each subset (**Figure 1D**; **Figures 2A, B**).

**Figure 2:**
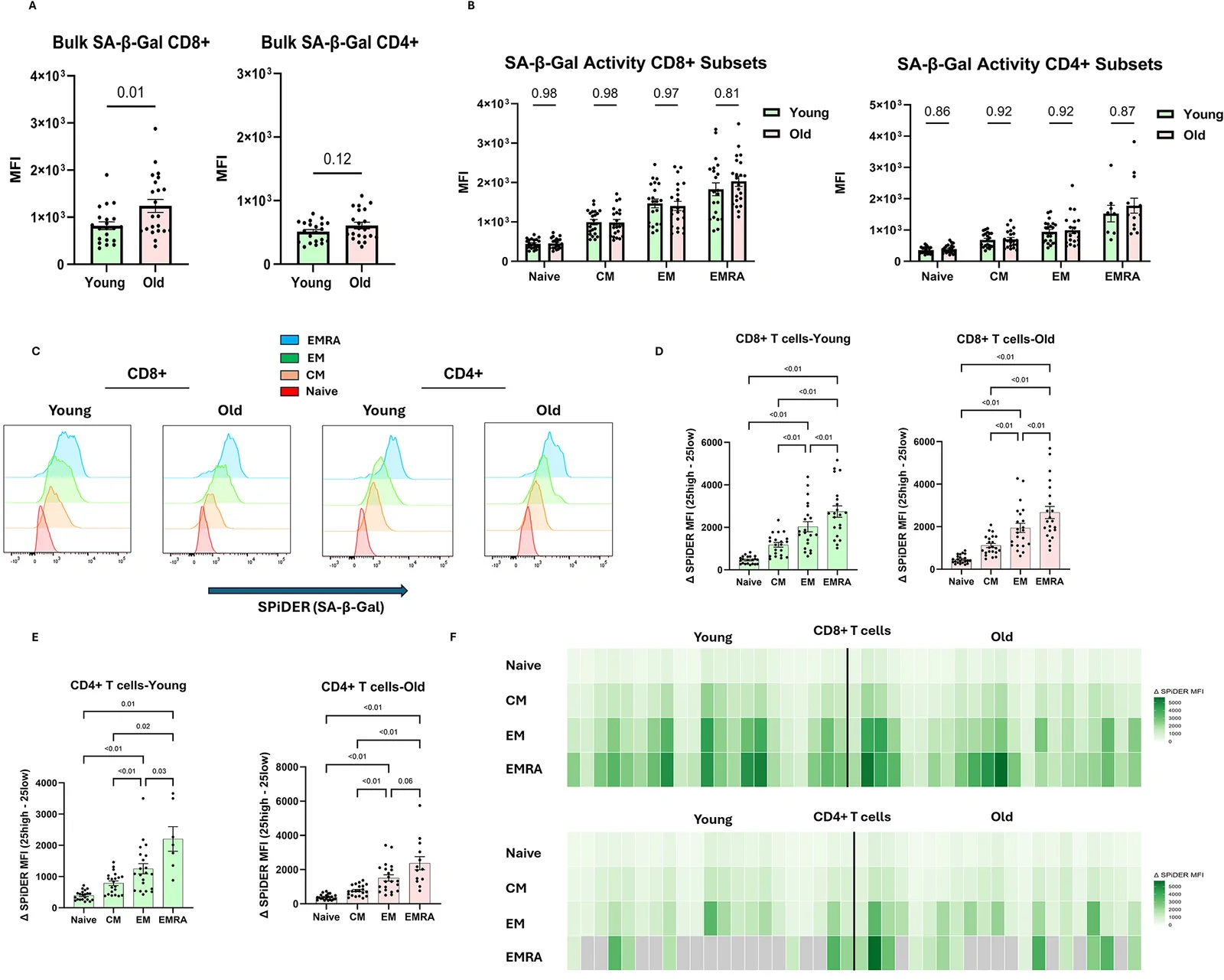
SA-β-Gal activity (SPiDER fluorescence) increases with T cell differentiation in both young and old donors. **(A)** SA-β-Gal activity (Median SPiDER fluorescence intensity) in CD8+ and CD4+ T cells in young (n=21) and old (n=22) donors. **(B)** SA-β-Gal activity (Median SPiDER fluorescence intensity) in CD8+ and CD4+ T cell subsets in young (n=21) and old(n=22) donors. **(C)** Histogram profiles reflecting SA-β-Gal activity (SPiDER fluorescence) in CD4+ and CD8+ **(D, E)** Bar graphs show ΔSPiDER MFI (highest 25% − lowest 25%) across naïve, CM, EM, and EMRA subsets of CD8+ (D) and CD4+ (E) T cells from young (n=21) and old (n=22) donors. **(F)** ΔSPiDER (25% High-25% Low) fluorescence matrix of the CD4+ and CD8+ subsets in young (n=20) and old (n=22) donors. Each column represents one donor, and color intensity reflects the relative SPiDER signal within each subset. Statistical significance was determined using an unpaired two-tailed Student’s t test **(A)**, multiple unpaired t-tests with Holm-Šidák’s correction **(B)**, repeated-measures two-way ANOVA with Tukey’s correction **(D)** and mixed-effects model with Tukey’s correction **(E)**. Bars indicate mean ± SEM. Figure created with BioRender.com.

Among all subsets, CD8_EM_ and CD8_EMRA_ displayed the broadest SA-β-Gal activity distributions between donors (**Figure 2C**), suggesting that the effector compartment contains substantial within-subset heterogeneity. To quantify this directly, we calculated ΔSPiDER MFI for each donor as the difference between the top and bottom quartiles of SPiDER fluorescence. ΔSPiDER increased with differentiation but not with age and reached its highest values in EM and EMRA populations across both lineages (**Figures 2D, E**; **Supplementary Figure S1A**). We therefore focused subsequent analyses on the CD8+ compartment because our prior work had already established SA-β-Gal profiling in human CD8+ T cells (15, 16) and CD8_EM_ and CD8_EMRA_ displayed the strongest heterogeneity.

Because CMV can shape the bulk CD8+ T-cell compartment by expanding differentiated memory populations (31, 32), which display higher SA-β-Gal activity than the naïve in our dataset, we asked whether CMV-specific cells were also preferentially enriched in the SA-β-Gal high fraction of CD8_EM_ and CD8_EMRA._ To assess the effect of CMV experience on SA-β-Gal expression, we measured known CMV-positive cells by tetramer analysis in the context of SA-β-Gal activity. CMV tetramer-positive cells were detected at similar frequencies in the SA-β-Gal low and SA-β-Gal high fractions of both CD8_EM_ and CD8_EMRA_ cells(**Supplementary Figures 1B–D**). These findings suggest that CMV-associated differentiation may contribute indirectly to higher SA-β-Gal activity at the bulk CD8+ T-cell level, but that SA-β-Gal heterogeneity within CD8_EM_ and CD8_EMRA_ cells is not primarily explained by enrichment of the assayed CMV-specific population.

### 3.2 SA-β-Gal activity stratification resolves recurrent senescence-associated phenotypic states within CD8_EM_ and CD8_EMRA_ cells

To determine whether SA-β-Gal quartile stratification captured recurrent phenotypic states rather than reflecting donor-specific shifts in SPiDER fluorescence within the low and high gates, we analyzed CD8_EM_ and CD8_EMRA_ cells using an independent panel of senescence-associated markers consisting of CD27, CD28, CD57, and CX3CR1. They were among the most differentially expressed genes between SA-β-Gal low and SA-β-Gal high bulk CD8+ T cells in our previously published RNA-seq dataset (15) (**Figure 3A**). We considered these markers of relevance in the context of SA-β-Gal activity because CD57 and CX3CR1 have been associated with terminal differentiation, inflammatory effector function, and senescence-like CD8 states, whereas loss of CD27 and CD28 marks reduced costimulatory/proliferative potential (11, 17, 33, 34). Consistent with this, CX3CR1 and CD57 increased with CD8+ T-cell differentiation, whereas CD27 and CD28 decreased, and these patterns were largely preserved across age groups (**Supplementary Figure 2A**).

**Figure 3:**
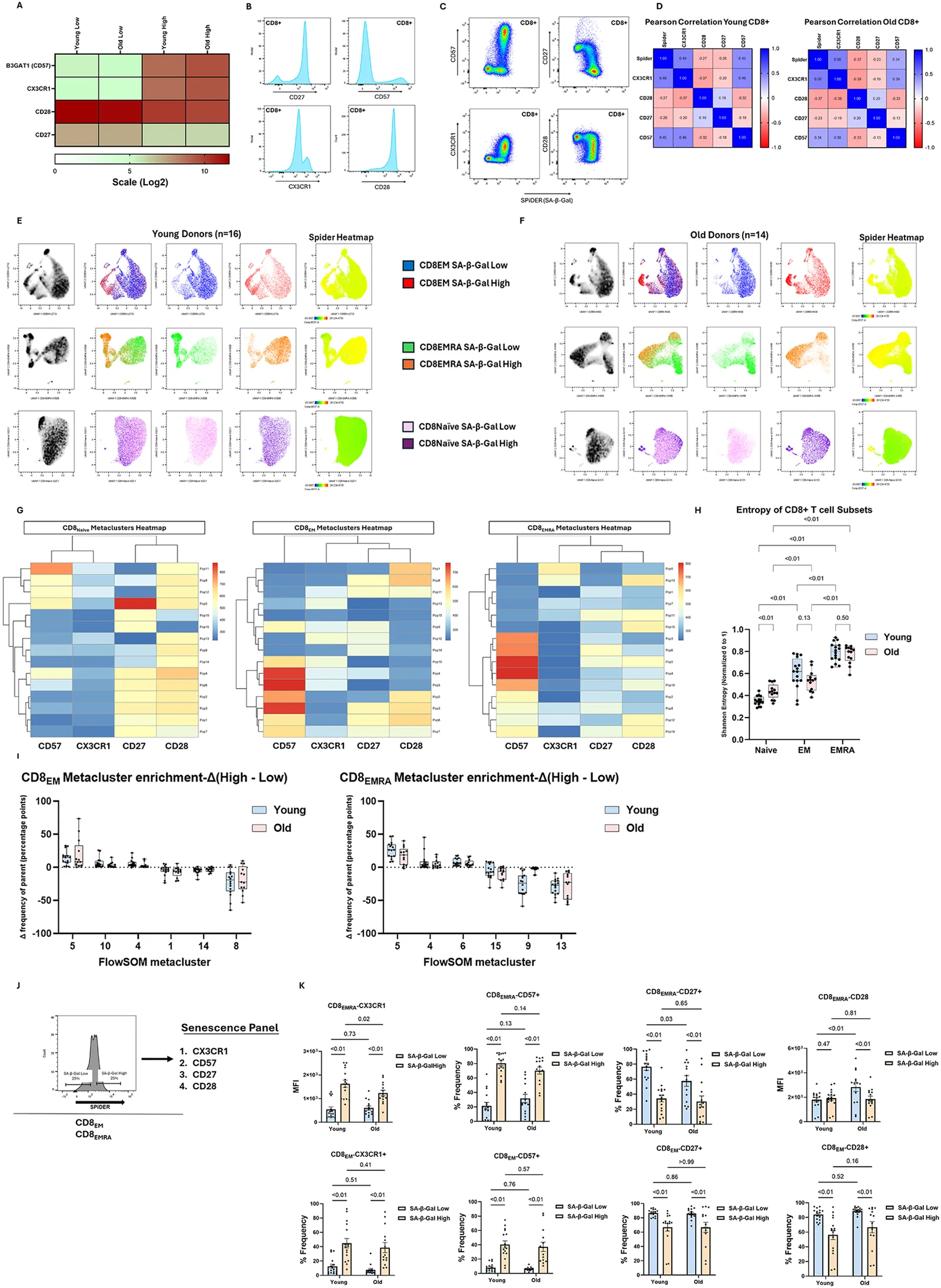
SPiDER (SA-β-Gal activity) stratification into quartiles resolves senescence-linked heterogeneity in effector CD8+ T-cells. **(A)** The heatmap illustrates the expression (bulk RNA-seq data) of senescence-related genes (CD57, CX3CR1, CD27, CD28) in bulk CD8+ T cells from young (n=3) and old (n=3) donors, stratified by SPiDER fluorescence (SA-β-Gal activity). **(B)** Representative histograms showing the expression of CD27, CD57, CX3CR1 and CD28 in CD8+ T cells. **(C)** Representative pseudo color dot plots showing the relationship between SPiDER (SA-β-Gal activity) signal and the expression of CD57, CD27, CX3CR1 and CD28 in CD8+ T cells. **(D)** Heatmaps show Pearson correlation coefficients between SPiDER signal and the expression of CX3CR1, CD28, CD27, and CD57 in bulk CD8+ T cells from young (n=16) and old (n=14) donors. Positive correlations are shown in blue and negative correlations in red. Coefficients describe single-cell associations in donor-concatenated datasets; no event-level significance tests were performed. **(E, F)** UMAP embeddings concatenated and pre-gated CD8_EM_, CD8_EMRA_ and CD8_Naive_ from young (n=16) and old (n=14) donors, generated using senescence-associated phenotyping markers (CD27, CD28, CD57, CX3CR1). For each subset, the leftmost plot shows the overall cell density, followed by overlays of SA-β-Gal low (Q1) and SA-β-Gal high (Q4) populations, and a heatmap showing SPiDER signal intensity across the UMAP projection. **(G)** Heatmaps summarizing the marker-expression profiles of FlowSOM metaclusters trained within CD8_Naive_, CD8_EM_, and CD8_EMRA_ subsets using CD27, CD28, CD57, and CX3CR1 as clustering inputs. **(H)** Donor-level phenotypic heterogeneity within CD8+ T-cell subsets quantified by normalized Shannon entropy of FlowSOM metacluster frequencies (K = 15) in Young (n=16) and old (n=13) donors. **(I)** Metacluster enrichment of SA-β-Gal High vs Low for CD8_EM_ and CD8_EMRA_ in young (n=16) and old (n=13) donors. Enrichment was calculated as Δ=f(SA-β-Gal High)−f(SA-β-Gal Low). **(J)** Representative SPiDER histogram showing the gating strategy used to gate the lowest 25% and highest 25% of SA-β-Gal signal within CD8_EM_ and CD8_EMRA_ cells, referred to as SA-β-Gal low and SA-β-Gal high. These populations were then interrogated for the expression of senescence-associated phenotypic markers, including CX3CR1, CD57, CD27, and CD28. **(K)** Quantification of CX3CR1, CD57, CD27, and CD28 expression in SA-β-Gal low and SA-β-Gal high populations within CD8_EM_ and CD8_EMRA_ cells from young (n=16) and old (n=14) donors. Graphs show either marker expression levels (median fluorescence intensity) or the frequency of marker-positive cells, as indicated for each panel. Boxplots **(H, I)** indicate the median and interquartile range. Bar graphs **(K)** indicate mean ± SEM. Statistical significance was determined using two-way repeated-measures ANOVA with Geisser-Greenhouse correction and Holm–Šídák multiple comparisons **(H)** and repeated-measures two-way ANOVA **(K)**. Figure created with BioRender.com.

Representative histograms show the expression distributions of these markers in CD8+ T cells (**Figure 3B**). We next examined how each marker related to SPiDER fluorescence at the single-cell level. Representative pseudocolor plots showed that cells with higher SPiDER signal were enriched for CD57 and CX3CR1 expression and were less enriched for CD27 and CD28 expression (**Figure 3C**). To quantify these relationships across age groups, we calculated Pearson correlations between SPiDER fluorescence and each marker in concatenated bulk CD8+ T-cell files from young and old donors. In the pooled bulk CD8+ datasets from both age groups, SPiDER fluorescence correlated positively with CD57 and CX3CR1 and negatively with CD27 and CD28 (**Figure 3D**). However, these correlations did not indicate a one-to-one relationship between SA-β-Gal activity and the individual senescence-associated markers. For example, despite the positive association between SA-β-Gal activity and CD57, SA-β-Gal high/CD57 low cells were also present (**Figure 3C**), indicating that these markers capture overlapping but non-interchangeable phenotypic features. Overall, the markers selected from our RNA-seq data also aligned with SA-β-Gal activity at the protein level, supporting their use as an independent phenotypic framework to interrogate SA-β-Gal-associated heterogeneity. We next asked whether SA-β-Gal low and SA-β-Gal high cells occupied different regions of the senescence-associated phenotypic landscape. To do this, we generated UMAP embeddings of concatenated CD8_EM_, CD8_EMRA_, and naïve CD8+ T cells from 16 young and 14 old donors using CD27, CD28, CD57, and CX3CR1 as input parameters while excluding SPiDER to avoid circularity, meaning that the same SA-β-Gal signal used to define low and high fractions was not allowed to drive the dimensionality reduction or clustering (**Figures 3E, F**). In both CD8_EM_ and CD8_EMRA_, SA-β-Gal low and SA-β-Gal high cells were not randomly intermixed, but instead showed preferential enrichment in different regions of the phenotypic landscape (**Figures 3E, F**). Thus, SA-β-Gal quartile stratification identified populations that were enriched in distinct regions of the UMAP-defined phenotypic space within CD8_EM_ and CD8_EMRA_ cells. By contrast, naïve cells showed much weaker spatial segregation, arguing against the possibility that quartile-based gating alone creates arbitrary intensity bins. These patterns were preserved across young and old donors, indicating that the overall organization of SA-β-Gal-associated heterogeneity is largely maintained across age groups (**Figures 3E, F**).

To confirm that these patterns were not driven by a small number of donors, we next applied FlowSOM, an unsupervised clustering method that groups cells into metaclusters based on marker-expression similarity. FlowSOM maps were generated from the same senescence-associated markers, again excluding SPiDER from the clustering inputs (**Figure 3G**). FlowSOM analysis showed broader phenotypic diversity in EM and EMRA than in naïve cells, as quantified by normalized Shannon entropy and donor-level metacluster distributions (**Figure 3H**; **Supplementary Figures 2B, C**). When the trained maps were applied to SA-β-Gal low and SA-β-Gal high quartile gates within each donor, specific metaclusters showed recurrent enrichment in either the SA-β-Gal high or SA-β-Gal low fractions of CD8_EM_ and CD8_EMRA_ cells (**Figure 3I**; **Supplementary Figure 2C**). Importantly, these directional enrichments were observed across multiple donors, indicating that SA-β-Gal high and SA-β-Gal low cells occupy reproducible, non-random regions of the effector phenotypic landscape. In contrast, naïve cells showed weaker redistribution between the two fractions, consistent with their lower degree of heterogeneity (**Supplementary Figure S2C**).

We then directly validated these phenotypic differences by quantifying CD27, CD28, CD57, and CX3CR1 within the bottom and top quartiles of SPiDER fluorescence in donor-matched CD8_EM_ and CD8_EMRA_ cells (**Figures 3J, K**; **Supplementary Figure 2D**). In both subsets, SA-β-Gal high cells displayed a more senescence-associated phenotype, characterized by increased CD57 and CX3CR1 and reduced CD27 relative to SA-β-Gal low cells. In CD8_EM_, the SA-β-Gal low fraction also retained a higher frequency of CD28+ cells, whereas CD28 differences in CD8_EMRA_ were more modest (**Figures 3J, K**; **Supplementary Figure S2D**). Direct comparisons between age groups revealed lower CX3CR1 expression in SA-β-Gal high CD8_EMRA_ cells from older donors as compared to younger donors (p = 0.02), whereas the other markers showed no statistically significant age differences within the SA-β-Gal high fractions of either effector subset (**Figure 3K**). Together, these data show that quartile-based SA-β-Gal stratification reproducibly enriched for specific CD27/CD28/CD57/CX3CR1-defined phenotypes within human CD8 effector subsets rather than arbitrary extremes of fluorescence.

### 3.3 SA-β-Gal stratification resolves distinct baseline granzyme-expression patterns within CD8_EM_ and CD8_EMRA_ cells

Granzymes are central components of CD8+ T-cell effector identity and have previously been linked to senescence-associated T-cell states (35–38). In fact, multiple studies using the senescence-associated markers described above have reported elevated Granzyme B (Gzm B) and granzyme A (Gzm A) levels in senescent T cells (34, 39–41). However, it has not been reported whether granzyme expression resolves in the same way within SA-β-Gal-defined effector states and whether that relationship is preserved across age. This question was particularly relevant in our system because CD8_EM_ and CD8_EMRA_ showed the greatest SA-β-Gal-defined heterogeneity (**Figure 2**) and therefore provided the best setting in which to test whether senescence-associated stratification also captures differences in cytolytic-potential organization. Because granzymes can reflect differentiation history and inflammatory bias in addition to cytotoxic potential, they provided an independent way to test whether the SA-β-Gal-defined states identified above were biologically structured rather than a pattern unique to our panel of senescence-associated markers. Using our previously published bulk RNA-seq dataset, we found that Gzm H, Gzm A, and Gzm B were among the most differentially expressed cytolytic transcripts between SA-β-Gal low and SA-β-Gal high CD8+ T cells from both young and old donors, with perforin (PRF1) and TNF-α also increased in the SA-β-Gal high group (**Figure 4A**).

**Figure 4:**
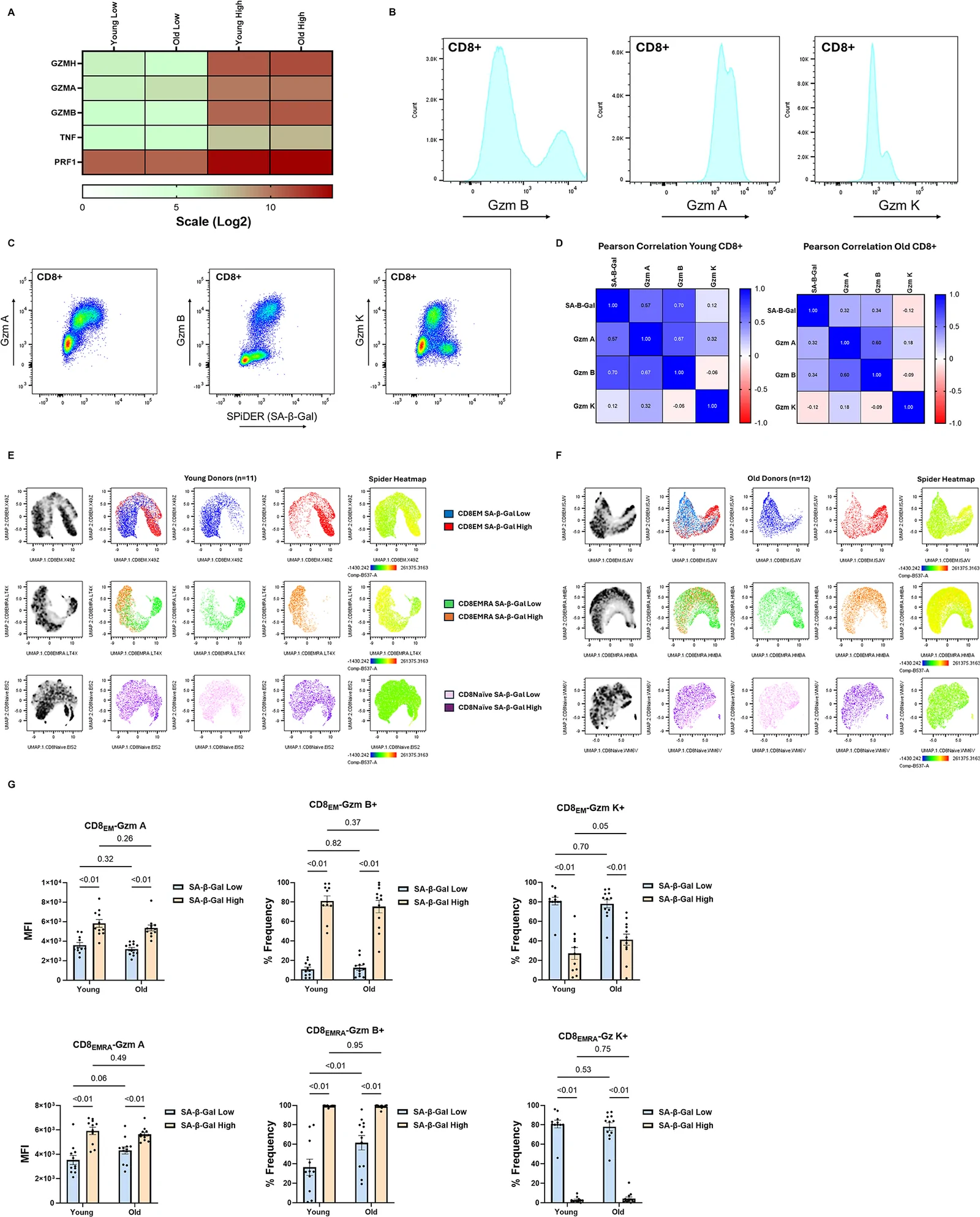
SA-β-Gal stratification reveals distinct granzyme programs within human CD8 effector subsets **(A)** The heatmap illustrates the expression (bulk RNA-seq) of cytolytic-associated genes (Gzm H, Gzm A, Gzm B, TNF, PRF) in bulk CD8+ T cells from young (n=3) and old (n=3) donors, stratified by SA-β-Gal activity. **(B)** Representative histograms showing the expression of Gzm B, Gzm A and Gzm K in CD8+ T cells. **(C)** Representative pseudo color dot plots showing the relationship between SPiDER (SA-β-Gal activity) signal and the expression of Gzm A, Gzm B and Gzm K in CD8+ T cells. **(D)** Heatmaps show Pearson correlation coefficients between SPiDER signal and the expression of Gzm A, Gzm B and Gzm K in CD8+ T cells from young (n=11) and old (n=12) donors. Positive correlations are shown in blue and negative correlations in red. Coefficients describe single-cell associations in donor-concatenated datasets; no event-level significance tests were performed **(E, F)** UMAP embeddings of concatenated and pre-gated CD8_EM_, CD8_EMRA_ and CD8_Naive_ from young (n=11) and old (n=12) donors, generated using granzyme markers (Gzm A, Gzm B and Gzm K). For each subset, the leftmost plot shows the overall cell density, followed by overlays of SA-β-Gal low (Q1) and SA-β-Gal high (Q4) populations, and a heatmap showing SPiDER signal intensity across the UMAP projection. **(G)** Quantification of Gzm A, Gzm B and Gzm K expression in SA-β-Gal low and SA-β-Gal high populations within CD8_EM_ and CD8_EMRA_ cells from young (n=11) and old (n=12) donors. Graphs show either marker expression levels (median fluorescence intensity) or the frequency of marker-positive cells, as indicated for each panel. Bar graphs **(G)** indicate mean ± SEM. Statistical significance was determined using repeated-measures two-way ANOVA **(G)**. Figure created with BioRender.com.

We therefore tested whether SA-β-Gal-associated heterogeneity within differentiated CD8+ T-cell subsets could also be resolved through a granzyme-based phenotypic framework. Although Gzm K transcripts were not detected in our bulk RNA-seq dataset, we included Gzm K in the flow cytometry panel because prior work identified Gzm K-expressing CD8+ T cells as an aging-associated population with clonal expansion, inflammatory features, and exhaustion-associated markers (40). At the protein level, Gzm A and Gzm B increased with CD8+ T-cell differentiation, whereas Gzm K showed a distinct distribution across subsets rather than a progressive increase with differentiation (**Supplementary Figure 3A**). These markers exhibited different expression patterns and relationships with SPiDER fluorescence within CD8+ T cells (**Figures 4B, C**). Descriptive Pearson analysis of the pooled bulk CD8+ datasets showed positive associations of SPiDER fluorescence with Gzm A and Gzm B in both age groups, whereas its association with Gzm K was weakly positive in young donors and weakly negative in older donors (**Figure 4D**). Together, these data suggested that high SA-β-Gal activity preferentially aligns with a granzyme program enriched for Gzm A/Gzm B rather than Gzm K.

To determine whether these relationships also resolved spatially within differentiated subsets, we generated UMAP embeddings of concatenated CD8_EM_, and CD8_EMRA_ and naïve CD8+ T cells from 11 young and 12 old donors using Gzm A, Gzm B, and Gzm K as input parameters and overlaid the SA-β-Gal low and SA-β-Gal high fractions (**Figures 4E, F**). As in the senescence-marker analysis, SPiDER was excluded from the UMAP inputs to avoid circularity. In both CD8_EM_ and CD8_EMRA_ cells, SA-β-Gal low and SA-β-Gal high cells preferentially occupied different regions of the granzyme-defined phenotypic manifold, indicating that SA-β-Gal-associated heterogeneity extends beyond senescence-associated markers and into the granzyme compartment. SA-β-Gal high cells preferentially aligned with Gzm A/Gzm B-enriched regions, whereas SA-β-Gal low cells retained greater representation in Gzm K-enriched regions (**Figures 4E, F**). Thus, the separation was not limited to senescence-associated markers but extended to a second, functionally relevant phenotypic axis. In contrast, naïve CD8+ T cells showed much weaker spatial segregation, consistent with the lower degree of heterogeneity observed in this subset. These patterns were preserved across young and old donors, indicating that SA-β-Gal quartile gating captures recurrent granzyme-associated states largely independently of age (**Figures 4E, F**).

We then directly validated these granzyme-defined states by quantifying Gzm A, Gzm B, and Gzm K within the bottom and top quartiles of SPiDER fluorescence in donor-matched CD8_EM_ and CD8_EMRA_ cells (**Figure 4G**; **Supplementary Figures 3B, C**). Because Gzm B and Gzm K showed bimodal distributions, they were quantified as the frequency of positive cells, whereas Gzm A was quantified by median fluorescence intensity (**Supplementary Figure 3C**). Using this approach, the SA-β-Gal high fraction in both CD8_EM_ and CD8_EMRA_ cells showed a higher frequency of Gzm B+ cells and increased Gzm A expression relative to the SA-β-Gal low fraction, whereas the SA-β-Gal low fraction retained a higher frequency of GzmK+ cells (**Figure 4G**). Within the SA-β-Gal high fraction, older donors showed a higher frequency of Gzm K+ CD8_EM_ cells than younger donors (p = 0.05), whereas the corresponding CD8_EMRA_ comparison showed no detected age difference (p = 0.75). Gzm A and Gzm B measurements showed no statistically significant age differences within the high fractions of either subset (**Figure 4G**). Together, these data show that quartile-based SA-β-Gal stratification reproducibly separates differentiated CD8+ T cells into populations with distinct granzyme programs, with SA-β-Gal high and SA-β-Gal low effector subsets occupying different positions within the granzyme landscape (**Figures 4E–G**). A similar inverse Gzm B-Gzm K relationship was observed using a published single-cell RNA-seq dataset from healthy controls and patients with multiple myeloma, supporting the broader relevance of these granzyme-defined states beyond our primary cohort (**Supplementary Figure 3D**) (25).

### 3.4 Nutrient-limited conditions increase stimulation-induced cell death in SA-β-Gal high CD8 effector subsets

We next asked whether the phenotypically distinct SA-β-Gal-defined states within CD8 effector subsets also differed in their susceptibility to cell death under conditions of combined metabolic and activation stress. This question was relevant for two reasons. First, although senescent cells in canonical non-immune systems often engage BCL-family-dependent pro-survival programs, these dependencies vary across senescent cell types. For example, senescent fibroblasts have been associated with persistent BCL-2 expression, whereas other senescent cell models rely more strongly on BCL-XL and BCL-W for survival (22, 23, 42, 43). Therefore, it should not be assumed that these survival programs apply uniformly across immune contexts. Second, senescent human CD8+ T cells undergo marked metabolic remodeling, including altered nutrient uptake, mitochondrial function, and metabolic flexibility (14, 44, 45), raising the possibility that nutrient-limited conditions could expose vulnerabilities that are not evident under resting conditions. Because any difference in survival would be most informative if it emerged only when cells were forced to balance activation with limited nutrient availability, we used a stress model of nutrient-limited conditions rather than a steady-state comparison. Having identified recurrent SA-β-Gal-associated phenotypic differences within effector subsets in both age groups, we focused the subsequent sorted-cell survival experiments on younger donors. Their higher estimated absolute CD8_EM_ counts also favored their use for experiments requiring sufficient cells from each sorted quartile (**Figure 1D**). Therefore, these experiments addressed state-associated responses to combined stimulation and nutrient limitation, rather than the effect of chronological age on cell-death susceptibility. In parallel, Ingenuity Pathway Analysis of our previously published bulk RNA-seq dataset (15) showed enrichment of apoptosis-related pathways in resting SA-β-Gal high CD8+ T cells relative to SA-β-Gal low cells (**Supplementary Figure 4**), providing an independent rationale to test whether SA-β-Gal-defined states differ in their death response under stress.

To test this directly, we used a strong-activation model based on PMA/ionomycin, building on prior studies showing that robust stimulation can induce T cell death and that this susceptibility is shaped by signaling and metabolic context (46–49). Because the frequency of CD28+ cells differed between the SA-β-Gal low and SA-β-Gal high fractions, with fewer CD28+ cells in the SA-β-Gal high fractions (**Figure 3K**), we used PMA/ionomycin rather than anti-CD3 and -28 stimulation to minimize confounding from differences in proximal TCR/CD28 signaling. We selected PMA/ionomycin here not as a physiological surrogate of antigen recognition, but as a controlled strong-activation model that standardizes downstream signaling across phenotypically distinct effector populations. To impose metabolic challenge, we coupled this stimulation model to nutrient-replete media with RPMI-1640 medium (without FCS) or a nutrient-deprived medium with Hank’s Balanced Salt Solution (HBSS), which provides magnesium, calcium and glucose but lacks the more complete nutrient composition of RPMI. HBSS-based conditions have been used to model nutrient-limited conditions and apoptosis in mammalian cells in the context of metabolic stress, autophagy, and amino acid limitation (50–53). In parallel, nutrient shortage has been shown to regulate apoptosis susceptibility in activated human T cells through BCL-2-family pathways (49, 54). Based on this rationale, we used HBSS as a controlled nutrient-limited condition to test whether SA-β-Gal low and SA-β-Gal high CD8_EM_ and CD8_EMRA_ cells differed in their survival response to PMA/ionomycin stimulation, while separating stimulation-induced death from baseline differences in survival under nutrient-limited conditions.

In our experiments, we measured cell death through loss of plasma membrane integrity using Cytotox, PI, or Zombie dyes, and monitored apoptotic signaling through caspase-3/7 activation and phosphatidylserine (PS) externalization detected using ApoTracker. Under RPMI culture conditions, stimulation had little effect on death kinetics in the SA-β-Gal low and SA-β-Gal high fractions in either CD8_EMRA_ or CD8_EM_ cells (**Figures 5A, B**; **Supplementary Figures 5A, B**). In contrast, culturing cells with HBSS resulted in a marked stimulation-induced death response in both effector subsets, and this response emerged earlier and more abruptly in the SA-β-Gal high effector fractions than in their SA-β-Gal low counterparts. In CD8_EMRA_ cells, stimulated SA-β-Gal high cells showed a clear increase in death relative to stimulated SA-β-Gal low cells across the 18 h time course, and donor-level analysis supported both an early divergence at 2 h and a greater integrated death response across the full assay window (**Figure 5B**). In CD8_EM_ cells, all three donors showed a greater stimulation-associated fold change in cell death in the SA-β-Gal high fraction at 2 h, although neither the 2-h comparison nor the 0–18-h AUC comparison reached statistical significance (P = 0.27 and P = 0.38, respectively; **Supplementary Figure 5B**). These findings provide exploratory evidence of an early difference between the fractions, with greater donor-to-donor variability over the full observation period. Thus, nutrient withdrawal and strong activation cooperated to expose an early death vulnerability in SA-β-Gal high effector cells, with the most sustained phenotype observed in CD8_EMRA_ cells.

**Figure 5:**
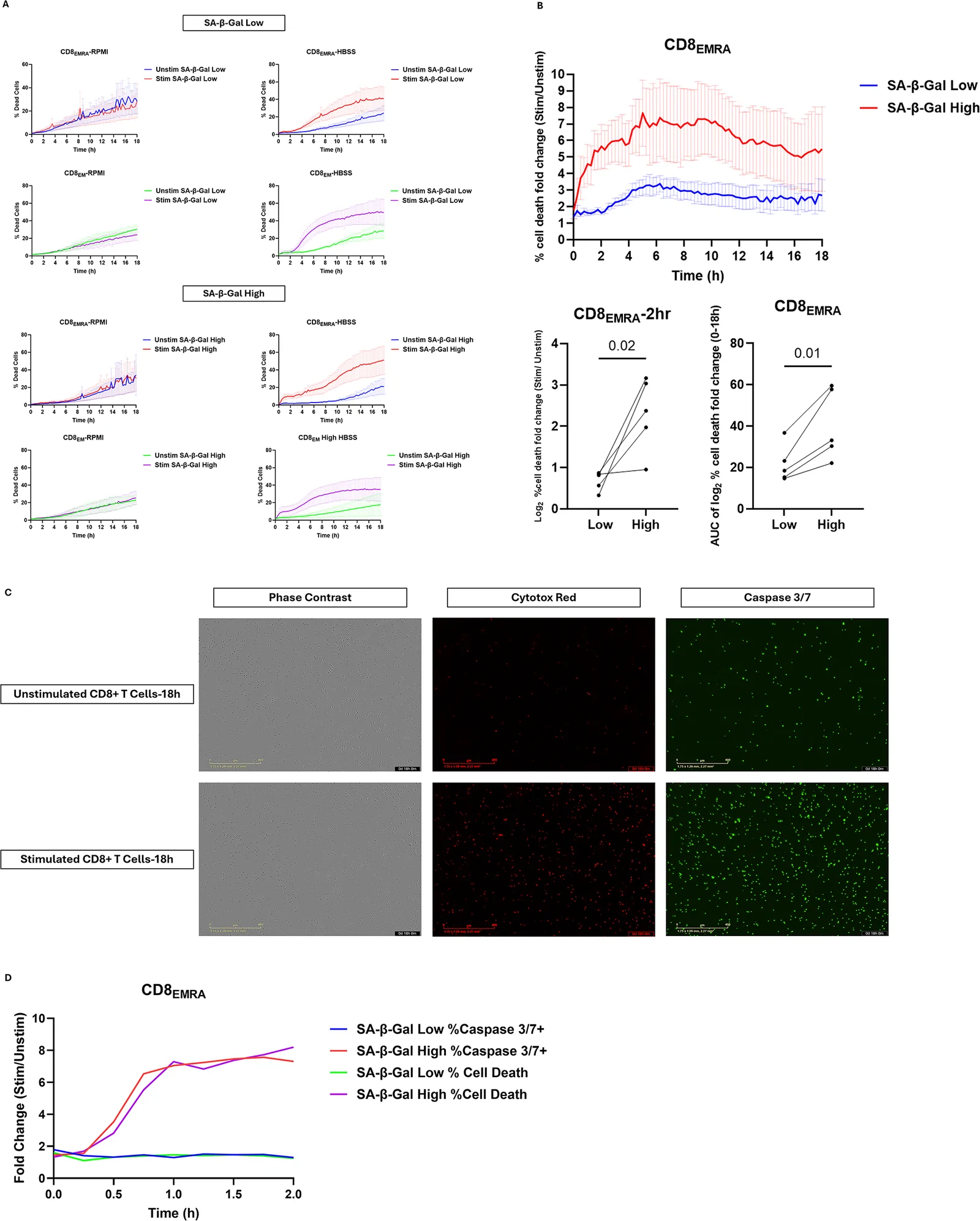
SA-β-Gal high cells undergo increased cell death following PMA and ionomycin stimulation under nutrient-limited conditions. **(A)** Sorted SA-β-Gal low (lowest 25%) and SA-β-Gal high (highest 25%) CD8_EMRA_ (n=3 in RPMI and 5 in HBSS) and CD8_EM_ cells (n=2 in RPMI and n=3 in HBSS) from individual donors were cultured in RPMI or HBSS in the presence (stim) or absence (unstim) of stimulation with PMA and ionomycin (500ng/ml each). Cell death was monitored over the common 0–18 h interval available for all included donors by propidium iodide (PI) uptake using the IncuCyte live-cell imaging platform. The upper panels show SA-β-Gal low cells and the lower panels show SA-β-Gal high cells. Curves show the arithmetic mean ± SEM across independent donors after averaging technical replicates within each donor. Stimulated and unstimulated conditions and SA-β-Gal low and high fractions were matched within donors, whereas donor availability differed between media. Individual donor measurements are provided in **Supplementary Table 1**. **(B)** The top panel shows the arithmetic mean ± SEM of the donor-specific fold change in cell death under stimulated relative to matched unstimulated conditions over 18 h (n = 5 donors). The bottom left and bottom right panels show paired donor-level comparisons of the log2 fold change in cell death at 2 h and the area under the curve, AUC, of the log2 fold change over 0 to 18 h, respectively. Each donor represents an independent experiment. **(C)** Representative IncuCyte images (10x) taken after an incubation time of 18h. Bulk CD8+ T cells were cultured in HBSS in the presence or absence of PMA and ionomycin stimulation and imaged after 18 h of incubation using the Incucyte live-cell imaging platform. Representative phase-contrast, Cytotox Red, and Caspase 3/7 fluorescence images are shown for unstimulated and stimulated conditions. **(D)** Sorted SA-β-Gal low, lowest 25%, and SA-β-Gal high, highest 25%, CD8_EMRA_ cells from a representative donor were cultured in HBSS in the presence or absence of PMA plus ionomycin, 500 ng/mL each. Cell death and apoptosis were monitored over time by Cytotox Red and Caspase-3/7 dye uptake using the IncuCyte live-cell imaging platform. Curves show the fold change in Cytotox-positive and Caspase-3/7-positive cells under stimulated relative to unstimulated conditions during 2 h. For IncuCyte assays, two technical replicates were used per donor per condition; images were acquired every 15 min, with four fields collected per well at each time point. Technical replicates were averaged before donor-level analysis. Statistical significance in panel B was determined by paired t test. Figure created with BioRender.com.

To determine whether this phenotype was accompanied by apoptotic signaling, we next monitored caspase-3/7 activity together with membrane-impermeant death probes during the same time course in the IncuCyte. In bulk CD8+ T cells, PMA/ionomycin stimulation in HBSS induced a coordinated increase in cell death and caspase-3/7 signal as illustrated by representative phase and fluorescence images obtained after 18 h (**Figure 5C**), while kinetic curves supported independent detection of the Cytotox and caspase channels (**Supplementary Figure 5C**). When the same approach was applied to sorted SA-β-Gal low and SA-β-Gal high effector cells, representative early kinetic curves again showed more rapid induction of both death and caspase-3/7 activity in the SA-β-Gal high fraction under nutrient-limited stimulation, in both CD8_EMRA_ and CD8_EM_ cells (**Figure 5D**; **Supplementary Figure 5D**). Together, these findings show that SA-β-Gal high CD8 effector cells are not uniformly more susceptible to death across conditions, but become selectively vulnerable when strong stimulation is imposed under nutrient limitation. This effect is strongest and most sustained in CD8_EMRA_ cells, while CD8_EM_ cells also display evidence of accelerated early death initiation, a pattern that was further supported by orthogonal flow-cytometric analysis in the subsequent figure (**Figure 6**).

**Figure 6:**
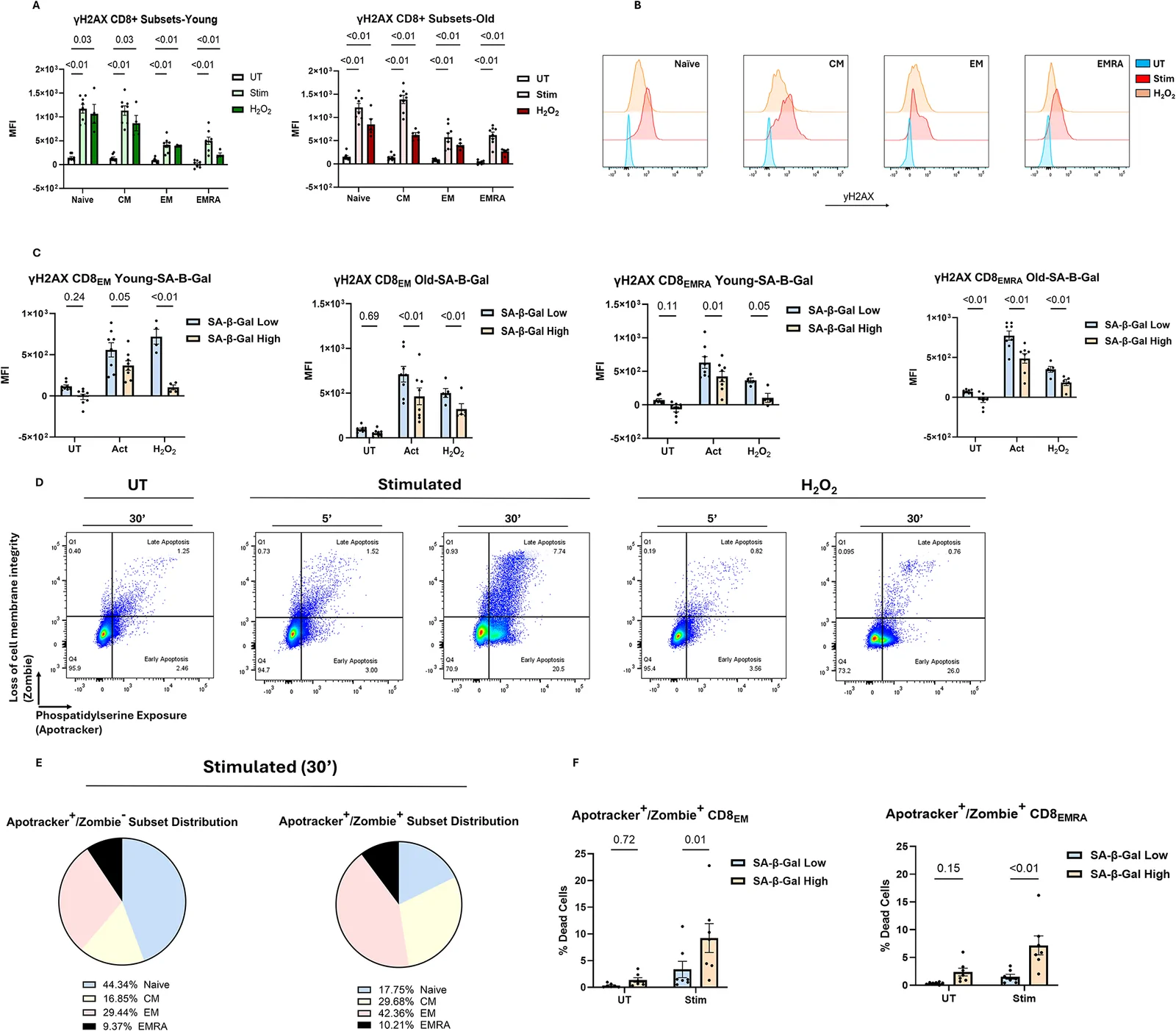
Stimulated SA-β-Gal high effector subsets show increased early cell death and lower γH2AX post-treatment signal than SA-β-Gal low subsets **(A)** γH2AX median fluorescence intensity (MFI) was measured in naïve, CM, EM, and EMRA CD8+ T-cell subsets from young and old donors under untreated (UT) conditions (young n=8, old n=8), after stimulation (Stim) with PMA (500ng/ml) and ionomycin (500ng/ml) (young n=8, old n=8) or following H_2_O_2_ (50 µM) (young n=4, old n=5) treatment as a positive control. **(B)** Representative histograms of young donor Y176 showing the expression of γH2AX in CD8+ T-cell subsets under untreated conditions (UT), after stimulation (Stim) with PMA (500ng/ml) and ionomycin (500ng/ml), or following H_2_O_2_ (50 µM) treatment as a positive control **(C)** Effector subsets from donors in **(A)** were stratified based on SA-β-Gal low (lowest 25%) and SA-β-Gal high (highest 25%) activity and γH2AX median fluorescence intensity (MFI) was measured under untreated conditions (UT), after stimulation (Stim) with PMA (500ng/ml) and ionomycin (500ng/ml), or following H_2_O_2_ (50 µM) treatment as a positive control. **(D)** Representative pseudo color dot plots of bulk CD8+ T cells showing phosphatidylserine exposure (Apotracker) and loss of membrane integrity (Zombie) under untreated (UT), stimulated with PMA (500ng/ml) and ionomycin (500ng/ml), or following H_2_O_2_ (50 µM) treatment at the indicated time points. Quadrants indicate viable cells, early apoptotic cells, and late apoptotic cells **(E)** Pie charts show the relative contribution of naïve, CM, EM, and EMRA CD8+ T-cell subsets within the Apotracker-positive Zombie-negative population (early apoptosis) and the Apotracker-positive Zombie-positive population (late apoptosis) after 30 min of stimulation. Values indicate the percentage of each subset within the corresponding apoptotic compartment. **(F)** The percentage of Apotracker-positive Zombie-positive cells was measured in SA-β-Gal low and SA-β-Gal high populations (n=7) within CD8_EM_ and CD8_EMRA_ cells under untreated (UT) conditions and following stimulation (stim) with PMA (500ng/ml) and ionomycin (500ng/ml). Statistical significance was determined using a mixed-effects model with Dunnett’s **(A)** and Šídák’s **(C)** multiple comparisons corrections, and two-way ANOVA with Šídák’s multiple comparisons correction **(F)** Bar graphs **(A, C, F)** indicate mean ± SEM. Figure created with BioRender.com.

### 3.5 Lower post-treatment γH2AX signal accompanies accelerated late cell death in SA-β-Gal high CD8 effector subsets

Because IncuCyte analysis showed that SA-β-Gal high CD8_EM_ and CD8_EMRA_ cells diverged most clearly from their SA-β-Gal low counterparts during the early phase of stimulation-induced death, we next examined γH2AX signal and apoptotic-state markers at 30 min. This early time point was chosen to capture stimulation-associated stress responses before later measurements became confounded by accumulated death and by the greater baseline survival of SA-β-Gal high cells in unstimulated HBSS conditions.

Phosphorylation of H2AX at Ser139 is an early chromatin-associated event in the DNA damage response and is widely used as a sensitive marker of DNA damage-associated signaling. In flow-cytometry assays, γH2AX is quantified as fluorescence signal intensity, providing a population-level readout of the magnitude of γH2AX induction at the single-cell level (55, 56). Moreover, in activated T cells γH2AX signaling has been linked to strong stimulation, providing a relevant framework for our PMA/ionomycin model (57). Therefore, we stimulated CD8_EM_ and CD8_EMRA_ with PMA/Ionomycin and measured cell death, via plasma membrane integrity and PS expression, within the SA-β-Gal high and low fractions. We included H_2_O_2_ as a positive control for DNA damage (58) to test whether the γH2AX differences associated with SA-β-Gal state were preserved beyond PMA/ionomycin stimulation.

Following PMA/ionomycin stimulation or H_2_O_2_ exposure, γH2AX signal was generally higher in naïve and CM cells than in EM and EMRA cells (**Figures 6A, B**). Within both effector subsets, SA-β-Gal high fractions exhibited lower post-treatment γH2AX signal than their SA-β-Gal low counterparts (**Figure 6C**), indicating that this pattern was not restricted to PMA/ionomycin stimulation. Comparisons of donor-matched changes from baseline also supported smaller γH2AX increases in SA-β-Gal high fractions in older-donor CD8_EM_ and CD8_EMRA_ cells after PMA/ionomycin stimulation and in young-donor CD8EM cells after H_2_O_2_ treatment (**Supplementary Figure 5E**). To study the lower post-treatment γH2AX signal observed in SA-β-Gal high cells relative to SA-β-Gal low cells in the context of cell death, we next quantified apoptotic-state progression using PS expression (Apotracker dye) and loss of membrane integrity (Zombie viability dye). Following PMA/ionomycin stimulation, bulk CD8+ T cells rapidly accumulated cells in the Apotracker+/Zombie+ compartment, whereas H_2_O_2_ treated cells were characterized by PS expression with limited zombie positivity (**Figure 6D**). Subset analysis showed that the ApoTracker+/Zombie+ compartment was composed predominantly of differentiated CD8+ subsets (**Figure 6E**). When SA-β-Gal low and SA-β-Gal high fractions were compared directly within CD8_EM_ and CD8_EMRA_ cells, the SA-β-Gal high populations were enriched in the Apotracker+/Zombie+ fraction relative to their SA-β-Gal low counterparts (**Figure 6F**). In the context of the lower γH2AX response observed in these same populations, these data argue against interpreting higher γH2AX in SA-β-Gal low cells as simply reflecting greater irreparable damage. Instead, combined with the IncuCyte data they support a model in which SA-β-Gal low effectors retain a stronger γH2AX-associated response under stress, whereas SA-β-Gal high effectors show a blunted response together with faster progression towards late cell death. This interpretation is further supported by the subset-level analysis (**Figures 6A, B**), where naïve cells displayed the strongest γH2AX response and were underrepresented in the late-death compartment relative to more differentiated subsets. Although these data are consistent with more effective stress handling in the SA-β-Gal low state, they do not by themselves distinguish between stronger damage sensing, a more sustained DDR, or more efficient downstream repair.

In this framework, the higher γH2AX signal intensity observed in SA-β-Gal low cells after PMA/ionomycin or H_2_O_2_ exposure indicates greater post-treatment γH2AX signal under acute stress. Because these same cells showed less death-marker positivity than SA-β-Gal high cells, this pattern is more consistent with a more intact stress-response state than with greater lethal damage. These data do not establish that SA-β-Gal low cells repair DNA damage more efficiently. Rather, they show that SA-β-Gal low cells show higher post-treatment γH2AX signal under acute stress, whereas SA-β-Gal high cells show lower post-treatment γH2AX signal together with increased death-marker positivity. We therefore interpreted γH2AX signal intensity here as an integrated DDR-associated signaling output rather than as a direct measure of DNA repair efficiency.

Increased reactive oxygen species (ROS)-mediated signaling is coupled to increased senescence, apoptosis signaling and caspase activation (59–61). Conversely, pharmacological inhibition of the mTORC1 pathway through rapamycin has been observed to lower mitochondrial membrane potential, oxygen consumption and reduce the production of mitochondrial ROS (62–64). Our metabolic studies (manuscript in preparation) have shown that under the same conditions tested here, SA-β-Gal high CD8_EM_ and CD8_EMRA_ cells displayed increased basal mitochondrial activity and stimulation-induced mitochondrial ROS, that was reduced by low-dose rapamycin (100nM). This motivated us to test whether rapamycin modulation of mitochondrial ROS was sufficient to alter the PMA/Ionomycin-associated death phenotype. To do this, we examined how rapamycin dose-dependent effects under the same PMA/ionomycin stimulation model affected ROS production and apoptosis. Under the conditions tested here, a higher-dose (10µM) rapamycin did not suppress PMA/ionomycin-induced mitochondrial ROS and instead increased it in both CD8_EM_ and CD8_EMRA_ cells. This effect was more pronounced in the SA-β-Gal high fractions than in the corresponding SA-β-Gal low fractions, resulting in a greater fold increase in mitochondrial ROS under stimulated conditions with 10 μM rapamycin pretreatment (**Supplementary Figures 6A–E**). Thus, SA-β-Gal high effector subsets remained the more ROS-prone state not only under stimulation alone, but also under the additional pharmacologic perturbation by rapamycin. Importantly, bulk CD8+ T-cell IncuCyte analysis showed that 100 nM rapamycin did not reduce stimulation-induced cell death despite reducing mitochondrial ROS (**Supplementary Figure 6F**). By contrast, the 10 μM rapamycin condition increased mitochondrial ROS and was associated with enhanced cell death in a representative bulk CD8+ T-cell assay (**Supplementary Figure 6G**). Together, these findings indicate that rapamycin-mediated effects on mitochondrial ROS and cell death are dose-dependent and argue against a simple model in which lowering mTORC1-associated mitochondrial ROS alone is sufficient to prevent stimulation-associated cell death.

## 4 Discussion

Most mechanistic frameworks used to define cellular senescence were developed in fibroblast systems, where senescence is classically described as a state of proliferative arrest accompanied by acquisition of a pro-inflammatory secretory phenotype and altered survival programs (65–68). However, whether this framework can be extrapolated to CD8+ T cells, regardless of differentiation status or chronological age, remained uncertain. In our system, SA-β-Gal activity tracked more closely with T cell differentiation than with chronological age. Older donors showed lower estimated absolute counts of CD8_naïve_ and CD8_EM_ T cells, whereas we detected no statistically significant difference in CD8_EMRA_ counts. Despite the decrease in CD8_EM_ counts, their relative frequency remained similar between age groups, while CD8_EMRA_ frequency increased in older donors. Consistent with previous reports of age-associated remodeling of the CD8+ T-cell compartment (26–28), these findings illustrate that relative enrichment of differentiated subsets can accompany contraction of other compartments (specially the CD8_naive_ compartment) and does not by itself establish absolute expansion. We therefore reason that this pattern is better interpreted as a consequence largely depending on the reduction of naïve CD8+ T cells, rather than the selective expansion of senescent effector subsets.

Although the age-associated increase in the relative frequency of differentiated CD8+ T-cell populations in our cohort largely reflected attrition of the naïve compartment rather than proportional absolute expansion of effector subsets, this compositional remodeling may nevertheless contribute to inflammaging (69). As the naïve compartment contracts, a greater fraction of the circulating CD8+ T-cell pool is composed of differentiated populations with increased inflammatory and secretory potential. Human CD8_EMRA_ cells have been shown to exhibit a p38-regulated senescence-associated secretory phenotype, while age-associated GzmK+ CD8+ T-cell populations can enhance inflammatory responses in non-immune cells (11, 40). Similarly, our previous studies showed that SA-β-Gal high CD8+ T cells, regardless of age, display similar inflammatory and secretory transcriptional programs together with reduced proliferative capacity (15, 16). Moreover, inflammatory signals such as IL-15 can promote the survival, proliferation, and effector phenotype of differentiated CD8+ memory cells, suggesting the potential for a feed-forward relationship in which age-associated inflammation favors the persistence or generation of inflammatory CD8+ T-cell states that may, in turn, further contribute to inflammaging (14, 34). This interpretation is particularly important given the gradual increase in SA-β-Gal activity with T-cell differentiation observed in our dataset.

Our data further support a model in which SA-β-Gal activity stratification resolves recurrent heterogeneity within human CD8+ effector subsets. By combining quartile-based SA-β-Gal gating with UMAP and FlowSOM analyses, we found that CD8_EM_ and CD8_EMRA_ cells were reproducibly resolved into recurrent phenotypic states defined by differences in senescence-associated markers, including CD57, CX3CR1, CD27, and CD28. Importantly, this organization was largely preserved across age groups. Collectively, these data indicate that SA-β-Gal-associated state structure is not an artifact of donor age and that SA-β-Gal high and SA-β-Gal low fractions reproducibly enrich for different regions of the UMAP space rather than follow a random distribution. Extending this framework to the granzyme compartment showed that SA-β-Gal high and SA-β-Gal low effector cells also occupy distinct granzyme-associated baseline phenotypes, with the former enriched for Gzm A/Gzm B-associated programs and the latter retaining greater representation of GzmK-associated states. Together, these findings indicate that SA-β-Gal captures structured effector-state diversity rather than simply reflecting a graded increase in lysosomal signal across differentiated cells. Although Mogilenko et al. identified Gzm K+ CD8+ T cells as an age-associated inflammatory population (40), our subset-resolved SA-β-Gal stratification showed that Gzm K+ cells are preferentially represented in the SA-β-Gal low fraction of CD8_EM_ and CD8_EMRA_ cells. This does not contradict the aging-associated enrichment of Gzm K+ CD8+ T cells. Rather, it suggests that Gzm K-associated aging and the SA-β-Gal high Gzm A/Gzm B-enriched state represent related but non-identical patterns of CD8+ T-cell remodeling. Consistent with this interpretation, older donors showed higher Gzm K+ frequencies within the SA-β-Gal high CD8_EM_ fraction, although Gzm K+ cells remained preferentially represented in the SA-β-Gal low fraction in both age groups (**Figure 4G**). Future studies combining proliferation assays with concurrent viability measurements and molecular senescence markers within donor-matched SA-β-Gal low and high CD8_EM_ and CD8_EMRA_ fractions could clarify how these phenotypic states relate to proliferative capacity.

An important functional implication of this study is that SA-β-Gal stratification identifies senescence-associated states with different cell-death responses within human CD8+ effector subsets. In many canonical non-immune models, senescent cells acquire pro-survival pathways that promote relative resistance to apoptosis. Our findings indicate that this paradigm does not fully apply to human effector T cells and instead depends strongly on biological context. In the absence of strong stimulation, SA-β-Gal high cells largely displayed greater resistance to cell death, even under nutrient-limited conditions, whereas coupling nutrient-limited conditions to PMA/ionomycin stimulation selectively exposed early vulnerability in the SA-β-Gal high state. Whether putting cells under stimulation in HBSS represents anything that mimics physiological conditions is doubtful. Under these conditions, SA-β-Gal high effector subsets exhibited greater early cell death, caspase-3/7 activation, lower post-treatment γH2AX signal, and more rapid progression into the apotracker+/zombie+ compartment as compared to their SA-β-Gal low counterparts. These findings are consistent with impaired stress adaptation in the SA-β-Gal high state, which is accompanied by a weaker DDR-associated response and more rapid progression toward cell death under combined metabolic and activation stress. These data support a model in which SA-β-Gal high state displays impaired stress adaptation under combined metabolic and activation stress (**Figure 7**). In this context, the higher γH2AX signaling observed in SA-β-Gal low cells is more consistent with a more competent DDR-associated response than with greater irreparable damage, because those same cells progressed less efficiently toward late death.

**Figure 7:**
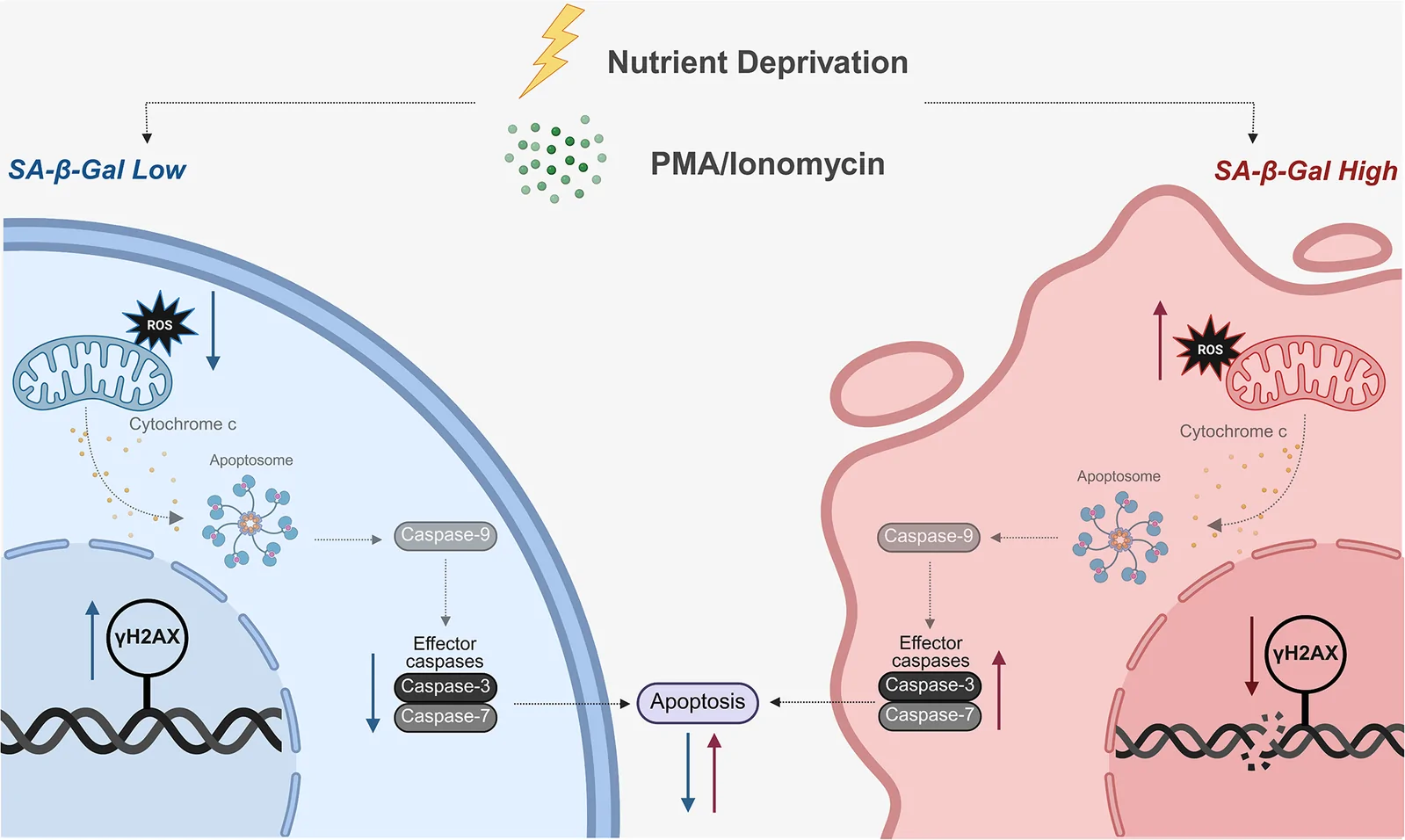
Schematic summarizing the proposed relationship between SA-β-Gal activity, γH2AX signaling, and apoptosis in CD8 effector subsets. Upon nutrient-limited conditions and PMA/ionomycin stimulation, SA-β-Gal low cells are proposed to sustain a stronger γH2AX-associated response and lower apoptotic signaling, whereas SA-β-Gal high cells display reduced γH2AX together with increased mitochondrial apoptotic signaling and caspase activation, culminating in increased apoptosis. Figure created with BioRender.com.

More broadly, our findings argue that senescence-associated features in T cells should not be interpreted solely through static markers of differentiation or baseline cytolytic phenotype, but as part of a broader state characterized by altered activation competence, stress handling, and survival under challenge. The fact that enhanced cell death emerged only under nutrient-deprived stimulation, whereas SA-β-Gal high cells were comparatively more resistant in the unstimulated setting, indicates that senescence-associated death susceptibility cannot be considered a fixed biological phenomenon. Rather, it appears to depend on the energetic demands imposed on the cell. This is particularly relevant given the growing links between metabolism, mitochondrial fitness, and T-cell effector function, as well as our broader findings that senescent-like CD8+ T-cell states display altered stress responses (70–74). The links between oxidative stress, lipid homeostasis and senescence described in cancer models provide a rationale for investigating the metabolic basis of these effector states (75, 76). Direct metabolic profiling would help distinguish specific metabolic alterations from the phenotypic and survival differences demonstrated here. Evidence linking immune-cell metabolism to impaired antitumor immunity and treatment resistance further motivates examining these states in metabolically restrictive disease environments (77). Whether the SA-β-Gal-associated survival differences observed here also occur in such settings remains to be established. At the same time, several limitations should be stated explicitly. First, PMA/ionomycin in HBSS is a strong stress model rather than a physiological surrogate of antigen recognition. Second, Bafilomycin A1 pretreatment was required for the live-cell SA-β-Gal staining protocol used here. Although the opposite survival patterns under stimulated and unstimulated conditions demonstrate context dependence, we cannot exclude the possibility that pretreatment altered the difference in stimulation-associated cell death between the SA-β-Gal low and high fractions. Third, γH2AX was used here as a DDR-associated readout and does not by itself distinguish between stronger damage sensing, a more sustained DDR response, or more efficient downstream repair. Fourth, although our data support accelerated cell death and an increased PMA/Ionomycin induced cell death in the SA-β-Gal high state, a detailed mechanistic explanation of this phenomenon in the context of potential divergences in the cell death pathways of each SA-β-Gal fraction remains unanswered. Within those limits, the present study identifies a context-dependent death-vulnerable effector state within human CD8+ T cells and provides a framework for testing how senescence-associated state organization influences immune function in metabolically restrictive environments.

The effects of rapamycin on oxidative stress are highly context dependent. Although rapamycin inhibits mTORC1, its downstream effects on mitochondrial function and ROS production can vary with dose and cellular context, with both reductions and increases in mitochondrial ROS having been reported (78, 79). Our supplementary rapamycin dose-response analysis further supports this idea and refines the interpretation of ROS in our cell death system. Although low-dose rapamycin reduced stimulation-induced mitochondrial ROS, it did not reduce cell death, indicating that ROS reduction alone is not sufficient to rescue stimulation-associated cell death. Conversely, higher-dose rapamycin increased mitochondrial ROS, with a stronger effect in the SA-β-Gal high state, and in a representative bulk assay this tracked with enhanced cell death. These findings argue against a simple model in which mTOR-linked modulation of ROS directly determines death outcome. Instead, they support a framework in which mitochondrial ROS contributes to the stressed SA-β-Gal high state, but is not by itself sufficient to explain commitment to stimulation-induced death. Future studies could determine whether differences in DNA-damage signaling, BCL-2-family regulation of apoptosis, or mTOR-dependent control of protein synthesis and autophagy contribute to the distinct survival responses of SA-β-Gal low and high effector cells. Defining these mechanisms may identify interventions that improve T-cell survival under metabolic stress (80–82).

## Data Availability

Publicly available datasets were analyzed in this study and are described at https://pubmed.ncbi.nlm.nih.gov/33939265/ and https://pmc.ncbi.nlm.nih.gov/articles/PMC10985429/. Donor-level cell-death measurements underlying **Figure 5A** and donor information are provided in **Supplementary Tables 1** and **2**, respectively. Additional data generated in this study are available from the corresponding author upon reasonable request.
