# Supplementary material for: SA-β-Gal stratification reveals senescence-associated heterogeneity and differential cell death susceptibility in human CD8 effector T cells
Source: Front Immunol. 2026 Oct 26;17:1985303. doi: 10.3389/fimmu.2026.1985303 (PMC13745467; doi:10.3389/fimmu.2026.1985303)
Supplement: Supplementary file 1 [file SupplementaryFile1.docx]

**Supplemental Figure 1: SA-β-Gal activity (SPiDER fluorescence) increases with T cell differentiation in both young and old donors, but effector subsets with high SA-β-Gal activity are not predominantly enriched in the CMV+ group.** (A) Bar graphs show ΔSPiDER median fluorescence intensity (25% High-25% Low) across naïve, CM, EM, and EMRA subsets from young (n=21) and old donors (n=22). Statistical significance was determined using repeated-measures two-way ANOVA with Tukey’s correction (A). Bar graphs (A) indicate mean ± SEM. (B) Representative flow cytometry plots showing tetramer staining in a CMV-negative unstained (US) control, a CMV-negative stained control, and a CMV-positive stained sample. Tetramer-positive events were identified within CD8+ T cells using Tetramer-PE and CD8-APC staining. (C) Donut charts showing the differentiation subset composition of CMV tetramer-positive CD8+ T cells from individual donors. Percentages indicate the relative distribution of naïve, CM, EM, and EMRA cells within the tetramer-positive population. (D) Proportion of CMV tetramer-positive events from individual donors within the SA-β-Gal low and SA-β-Gal high fractions of CD8_EM_ and CD8_EMRA_ cells. Percentages indicate the relative contribution of tetramer-positive cells within each SA-β-Gal-defined population.

**Supplemental Figure 2: SPiDER (SA-β-Gal activity) stratification into quartiles resolves senescence-linked heterogeneity in effector CD8+ T-cells.** (A) Bar graphs showing the expression (represented as median fluorescence intensity) of senescence-associated markers CX3CR1, CD57, CD27 and CD28 across different subsets of young (n=16) and old (n=14) donors. (B) Boxplots showing the metacluster composition (% Frequency) of naïve, EM and EMRA in self-organized maps generated using senescence-associated markers (CD27, CD28, CD57 and CX3CR1). The trained self-organizing maps were generated on concatenated, per-donor downsampled events within naïve, EM and EMRA using CD27, CD28, CD57, and CX3CR1 as clustering inputs (SA-β-Gal excluded). The trained map was applied to each donor to quantify metacluster composition. (C) Heatmap showing donor-level differences in metacluster frequency between SA-β-Gal high and SA-β-Gal low populations within CD8_EM_, CD8_EMRA_, and CD8_Naive_ cells using the self-trained maps created as described in (B). Values are expressed as Δ (SA-β-Gal high minus SA-β-Gal low), with positive values in red and negative values in blue. Each row corresponds to a metacluster and each column to an individual donor. (D) Overlay histograms show the expression of CD57, CD28, CX3CR1, and CD27 in SA-β-Gal high and SA-β-Gal low populations within CD8_EM_ and CD8_EMRA_ subsets. Statistical significance was determined using multiple unpaired t-tests with Holm-Šídák correction (A). Bar graphs (A) indicate mean ± SEM and boxplots (B) indicate the median and interquartile range.

**Supplemental Figure 3: SA-β-Gal stratification reveals distinct granzyme programs within human CD8 effector subsets .** (A) Bar graphs showing the expression (represented as median fluorescence intensity) of Gzm A, Gzm B and Gzm K across different subsets of young (n=11) and old (n=12) donors. (B) histogram representing the gating strategy applied in the CD8_EM_ and CD8_EMRA_for comparing gzm in the SA-β-Gal low (1^st^ quartile) vs SA-β-Gal high (4^th^ quartile). (C) Overlay histograms show the expression of Gzm A, Gzm B and Gzm K in SA-β-Gal high and SA-β-Gal low populations within CD8_EM_ and CD8_EMRA_ subsets (D) Volcano plots showing the top differentially expressed genes in CD8+ TEM (CD3+ CD8+ CD57- CCR7-) and CD8+ TTE (CD3+ CD8+ CD57+) cells from healthy controls and patients with multiple myeloma, derived from published single-cell RNA-seq data (Favaloro et al., 2024). The x-axis shows average log2 fold change and the y-axis shows statistical significance as -log10 p value. Highlighted genes include Gzm B, Gzm A, and Gzm K in CD8+ TEM cells, and Gzm K, Gzm A, Gzm H, and Gzm B in CD8+ TTE cells. Statistical significance was determined using multiple unpaired t-tests with Holm-Šídák correction (A). Bar graphs indicate mean ± SEM.

**Supplemental Figure 4:** Canonical apoptotic pathways were tested for over-representation of genes differentially expressed in SA-β-Gal high versus SA-β-Gal low CD8⁺ T cells from bulk RNA-seq. Bar length indicates enrichment significance (−log₁₀ p, Fisher’s exact test); the vertical reference line denotes the reporting threshold (−log p = 1.3). Bar color indicates IPA activation z-score interpreted in the direction of SA-β-Gal high: orange (z > 0, pathway predicted activated in SA-β-Gal high), blue (z < 0, predicted inhibited), unfilled (z = 0), gray (no computable activity pattern). Pathways are ranked by significance. Analysis performed in QIAGEN IPA.

**Supplemental Figure 5: Stimulated SA-β-Gal high effector subsets undergo increased early cell death and exhibit exhibit lower post-treatment γH2AX signal.** (A) Sorted SA-β-Gal low, lowest 25%, and SA-β-Gal high, highest 25%, CD8_EMRA_ cells, n = 5 donors, and CD8_EM_ cells, n = 3 donors, were cultured in HBSS without stimulation or under PMA/Ionomycin (500ng/ml) stimulation. Cell death was monitored over time by propidium iodide (PI)/cytotox red uptake using the IncuCyte live-cell imaging platform. Curves compare the % cell death in SA-β-Gal low and high for CD8_EM_ and CD8_EMRA_ cells. Each donor represents an independent experiment. (B) For sorted CD8 effector memory SA-β-Gal low and high cells, the top panel shows the arithmetic mean ± SEM of donor-specific fold changes in cell death under PMA/ionomycin stimulation (500 ng/mL each) relative to matched unstimulated conditions over 18 h (n=3 donors). The top panel shows the arithmetic mean ± SEM of the donor-specific fold change in cell death under stimulated relative to matched unstimulated conditions over 18 h (n = 3 donors). The bottom left and right panels show paired donor-level comparisons of the log2 fold change in cell death at 2 h and the AUC of the log2 fold change over 0 to 18 h, respectively. Each donor represents an independent experiment. (C) Bulk CD8+ T cells of an independent donor were cultured in HBSS and monitored over 18 h by Incucyte live-cell imaging using Cytotox Red and Caspase 3/7 Green reagents under unstimulated and stimulated conditions with PMA and Ionomycin (500ng/ml each). The upper panel shows the percentage of Cytotox-positive cells (represented as dead cells), whereas the lower panel shows the percentage of Caspase 3/7-positive cells over time. These data demonstrate that the signal detected in the red Cytotox channel is not driven by background fluorescence from the green Caspase 3/7 reagent, and conversely, that the Caspase 3/7 signal is not due to spillover from the Cytotox channel, supporting independent detection of cell death and apoptosis. (D) Sorted SA-β-Gal low, lowest 25%, and SA-β-Gal high, highest 25%, CD8_EM_ cells from a representative donor were cultured in HBSS in the presence or absence of PMA/Ionomycin (500 ng/mL) each. Cell death and apoptosis were monitored over time by Cytotox Red and Caspase-3/7 dye uptake using the IncuCyte live-cell imaging platform. Curves show the fold change in Cytotox-positive and Caspase-3/7-positive cells under stimulated relative to unstimulated conditions during 2 h. For IncuCyte assays, two technical replicates were used per donor per condition; images were acquired every 15 min, with four fields collected per well at each time point. Technical replicates were averaged before donor-level analysis. (E) Baseline-adjusted γH2AX responses in SA-β-Gal low and high CD8_EM_ and CD8_EMRA_ fractions after PMA/ionomycin stimulation or H₂O₂ treatment. ΔγH2AX MFI was calculated by subtracting each donor’s matched untreated MFI from the corresponding treated MFI within each subset and SA-β-Gal fraction. PMA/ionomycin: n = 8 young and 8 older donors; H₂O₂: n = 4 young and 5 older donors. Points represent individual donors; bars show mean ± SEM. Statistical significance in panel B and E were determined by paired t test.

**Supplemental Figure 6: Rapamycin-mediated effects on mitochondrial ROS and cell death are dose dependent but lowering mTORC1-associated mitochondrial ROS alone is not sufficient to prevent the stimulation-associated cell death.** (A) Representative histograms showing the effect of rapamycin (1hr pretreatment) at different concentrations on PMA/Ionomycin (500ng/ml) induced mitochondrial ROS production in CD8_EM_ an CD8_EMRA_. (B-C) Bar graphs showing the effect of rapamycin at different concentrations on PMA/Ionomycin (500ng/ml) induced mitochondrial ROS production in SA-β-Gal Low and SA-β-Gal High CD8_EM_ and CD8_EMRA_ cells (n = 10). (D-E) Bar graphs showing the fold increase in mitochondrial ROS produced by SA-β-Gal Low and SA-β-Gal High CD8_EM_ and CD8_EMRA_ cells in B and C under stimulated conditions with 10uM rapamycin pretreatment relative to stimulated conditions. (F-G) IncuCyte time-course plots showing the effect of rapamycin (1hr pretreatment) on PMA/ionomycin (500ng/ml) induced cell death (% PI+ cells) in bulk CD8+ T cells over time. (H) Representative histograms showing the effect of rapamycin (1hr pretreatment) at different concentrations on PMA/Ionomycin (500ng/ml) induced mTORC1 measured by S6 phosphorylation (pS6). Tables show the median fluorescence intensity (MFI) for each of the conditions. Error bars in the bar graphs represent the mean ± SEM and ± SD in the IncuCyte time-course plots. Statistical significance was determined using paired t-tests (D, E) and repeated-measures one-way ANOVA(B, C) with multiple comparisons and Šidák’s correction. 2 technical replicates with a cell concentration of 5x10^4^/well were used in the IncuCyte assay and images were taken every 15-30’ over a 14-24h time course period
